# Diversity, distribution patterns, and fauno-genesis of the millipedes (Diplopoda) of mainland China

**DOI:** 10.3897/zookeys.930.47513

**Published:** 2020-04-28

**Authors:** Sergei I. Golovatch, Weixin Liu

**Affiliations:** 1 Institute for Problems of Ecology and Evolution, Russian Academy of Sciences, Leninsky pr. 33, Moscow 119071, Russia Institute for Problems of Ecology and Evolution, Russian Academy of Sciences Moscow Russia; 2 Department of Entomology, College of Agriculture, South China Agricultural University, 483 Wushanlu, Guangzhou 510642, China South China Agricultural University Guangzhou China

**Keywords:** continental China, Diplopod fauna, zoogeography

## Abstract

Based on all available information, 339 species from 71 genera, 26 families, and eleven orders of Diplopoda have hitherto been recorded from mainland China, the fauna thus being very rich, albeit far from completely known, comprising various zoogeographic elements and populating very different environments. Diplopods mainly occur in various woodlands, in caves, and high in the mountains. Most species (> 90 %, usually highly localised, including 160 cavernicoles), 18 genera, and one family are strictly endemic to continental China. Mapping not only the horizontal, but also the vertical distributions of Diplopoda in China shows the bulk of the fauna to be expectedly restricted to forested lowland and mountain biomes or their remnants. Yet some Chordeumatida, Callipodida, Polydesmida, Julida, and even Spirobolida seem to occur only in the subalpine to alpine environments and thus may provisionally be considered as truly high-montane. The long-acknowledged notions of China being a great biogeographic zone transitional between the Palaearctic and Oriental regions generally find good support in millipede distributions, in particular at the higher taxonomic levels (generic, familial, and ordinal). While the Palaearctic/Holarctic components expectedly dominate the fauna of the northern parts of the country, the Oriental ones prevail in its south and along the Pacific coast. Both realms are increasingly mixed and intermingled towards China’s centre. However, in addition to the above traditional views, based on distribution patterns alone, southern China seems to harbour a rather small, but highly peculiar faunal nucleus or origin centre of its own, whence Himalaya, Myanmar, Thailand, Indochina and/or Taiwan could have become populated by younger lineages. The millipede fauna of continental China is thus a tangled mixture of zoogeographic elements of various origins and ages, both relict and more advanced. The few anthropochores must have been the latest faunal “layer” to populate China.

## Introduction

Millipedes (Diplopoda) form a highly diverse, yet strongly understudied arthropod class with > 11,000 described species (Minelli 2015). Apparently, only ca. 20 % of the global species diversity of millipedes are currently known, with the actual number of species being estimated between 50,000 and 80,000 species (Minelli and Golovatch 2013). Being mainly represented by mesophilous forest-dwelling detritivores, millipedes have long been recognised as playing important ecological roles, mostly in temperate and tropical land ecosystems where their diversity is especially pronounced (Golovatch and Kime 2009).

The class encompasses 16 extant orders, 140+ families, and ca. 2,000 genera (Minelli and Golovatch 2013), while the distributions of higher taxa fully agree with the major biogeographic divisions of Earth into the Holarctic (Palaearctic + Nearctic), Afrotropical, Oriental, Neotropical and Australian regions which are accepted since Alfred Russel Wallace and Joseph Dalton Hooker. Antarctica is completely devoid of diplopods, whereas the Oriental Region appears to be the sole one to harbour all 16 orders. Being very ancient (Silurian, early Palaeozoic) and diverse taxonomically, widespread (present on all continents except Antarctica), virtually fully terrestrial (even fossils show spiracles), poorly vagile (with highly limited dispersal capacities) and highly limited in compensatory ecological faculties (strongly restricted by a single limiting ecological factor even if the others are favourable), Diplopoda have long been considered as an exemplary group for biogeographic studies and reconstructions (e.g. Shelley and Golovatch 2011).

China has long been considered as a huge territory lying between and linking the Palaearctic and Oriental realms, with very considerable areas of southern China representing not only a marked transitional zone (e.g. Wulf 1944; Zherikhin 2003; Holt et al. 2012), but also the largest karst belt of the world particularly rich in cavernicoles, including millipedes (Golovatch 2015a). Continental China as conventionally understood here includes Hainan and Hong Kong but excludes Taiwan. The territory in question covers ca. 9,326 million sq. km, spanning ca. 5,500 km from north to south and ca. 5,200 km from west to east. China’s topography is very complex. The outline descends step by step from west to east: mountains, high plateaus and hilly land prevail and take up nearly 70 % of the total area, with deserts also located in the west, but mostly plains, deltas and hills in the east. The climates are likewise varied, ranging from sharply continental in the north, through temperate in the middle, to monsoon subtropical and tropical in the south, with a warm humid influence along the eastern sea coasts (https://en.wikipedia.org/wiki/Geographic_information_systems_in_China).

**Figure 1.**
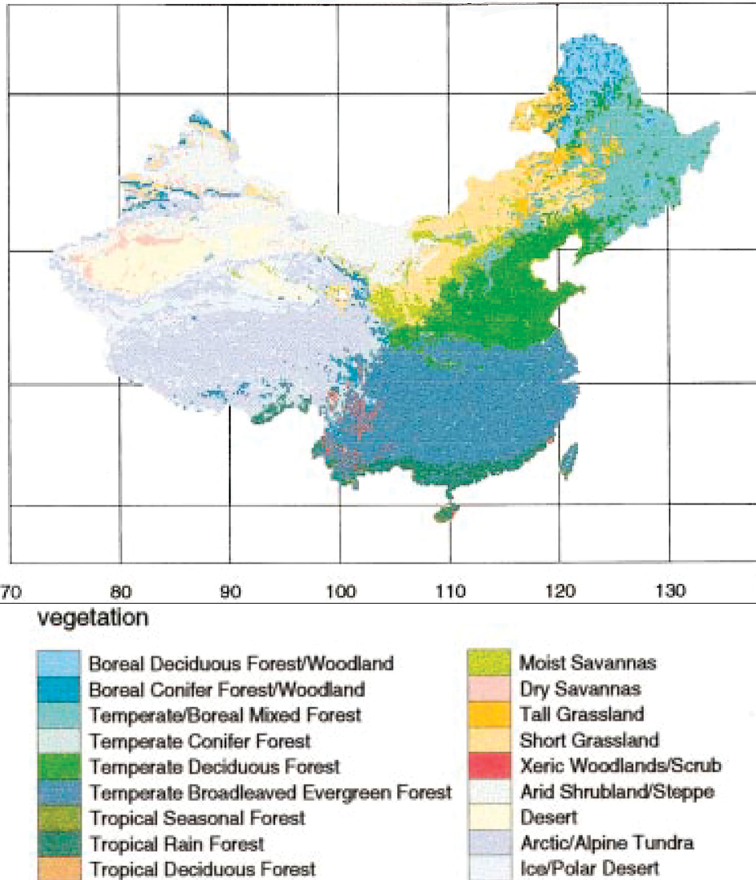
Nature zonation and the main biomes of China (after Ni et al. 2000).

China with its highly varied climates and relief (ca. 70 % national land area being mountains or plateaus) is exceptionally rich in ecological conditions and it supports as many as 18 natural latitudinal belts or biomes (Ni et al. 2000). They range from Polar desert and Alpine tundra in Tibet, through grasslands (savanna, steppe) or desert in the northern parts, to various woodlands (scrub, boreal forest, temperate forest, tropical forest etc.) (Fig. 1). Nature zonation is generally well-expressed, forested biomes prevailing in total area and forming a succession of boreal forest in the north, through temperate (conifer, deciduous and evergreen), to tropical rainforest in the far south. Altitudinal zonation follows the same general pattern which varies depending on location and grows increasingly complex from seven vegetation or eco-geographic belts in the Tianshan Mountains in the northwest or Tibetan Plateau in the southwest to 14 in Yunnan in the south (review by Zhang et al. 2004).

Even though the millipede fauna of China enjoys a very long history of taxonomic study, dating back to 1833 (Wang and Mauriès 1996), it still remains far from well-known. Based on all available information, 339 species from 71 genera, 26 families, and eleven orders of Diplopoda have hitherto been recorded from mainland China (Table 1), but there can be no doubt that our review will soon be out of date.

The present paper is an attempt not only to summarise the Chinese species list (as of the end of 2019), but also to provide an analysis of the distribution patterns revealed, both altitudinal and horizontal, and to hypothesise the main sources, routes and stages of fauno-genesis. A very similar approach has recently been applied to treating the millipedes of the Himalaya (Golovatch and Martens 2018).

## Materials and methods

Only described species and published records are considered in our paper, while dubious taxa and those not identified to the species level have been omitted from both checklist and bibliography.

**Figure 2.**
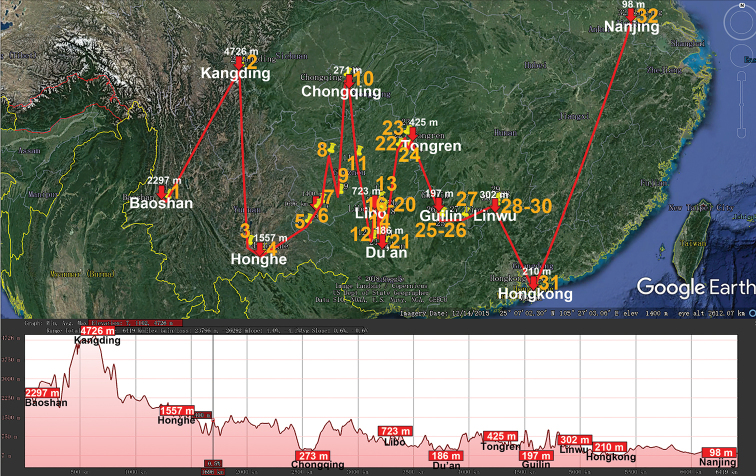
Distribution of the family Glomeridae, genus *Hyleoglomeris* in mainland China. Red lines show the transect Baoshan – Kangding – Honghe – Chongqing – Libo – Du’an – Tongren – Guilin – Linwu – Hong Kong – Nanjing, along which the elevations are crudely indicated below. **1***H. albicorporis***2***H. sinensis***3***H. reducta***4***H. maculata***5***H. heshang***6***H. gudu***7***H. rhinceros***8***H. nigu***9***H. getuhensis***10***H. aschnae***11***H. yinshi***12***H. grandis***13***H. eusulcata***14***H. xueju***15***H. wuse***16***H. qiyi***17***H. curtisulcata***18***H. mulunensis***19***H. kunnan***20***H. mashanorum***21***H. baxian***22***H. variabilis***23***H. multistriata***24***H. generalis***25***H. rukouqu***26***H. xuxiakei***27***H. lii***28***H. xia***29***H. youhao***30***H. tiani***31***H. bicolor*, **32***H. emarginata*.

**Figure 3.**
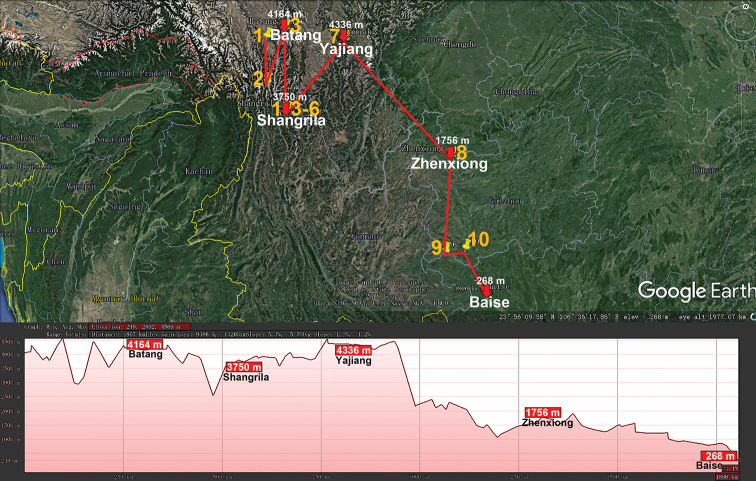
Distribution of the family Paracortinidae, genus *Paracortina* in mainland China. Red lines show the transect Batang – Shangrila – Yajiang – Zhenxiong – Baise, along which the elevations are crudely indicated below. **1***P. viriosa***2***P. serrata***3***P. thallina***4***P. carrinata***5***P. leptoclada***6***P. stimula***7***P. voluta***8***P. chinensis***9***P. yinae***10***P. zhangi*.

**Figure 4.**
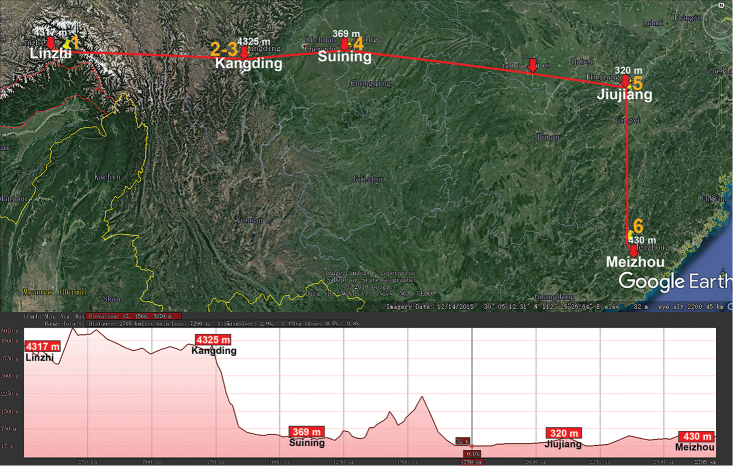
Distribution of the family Julidae, genus *Nepalmatoiulus* in mainland China. Red lines show the transect Linzhi – Kangding – Suining – Jiujiang – Meizhou, along which the elevations are crudely indicated below. **1***N. tibetanus***2***N. brachymeritus***3***N. rhaphimeritus***4***N. polyakis***5***N. fraterdraconis***6***N. eulobos*. *Nepalmatoiulus yunnanensis* is not mapped because no exact locality in Yunnan is known.

**Figure 5.**
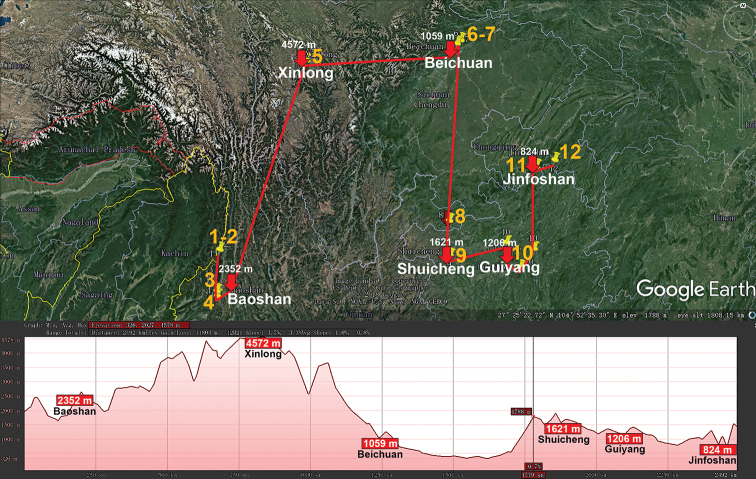
Distribution of the family Megalotylidae, genus *Nepalella* in mainland China. Red lines show the transect Baoshan – Xinlong – Beichuan – Shuicheng – Guiyang – Jinfoshan, along which the elevations are crudely indicated below. **1***N. kavanaughi***2***N. pianma***3***N. magna***4***N. griswoldi***5***N. marmorata***6***N. lobata***7***N. grandoides***8***N. grandis***9***N. caeca***10***N. troglodytes***11***N. jinfoshan***12***N. wangi*.

**Figure 6.**
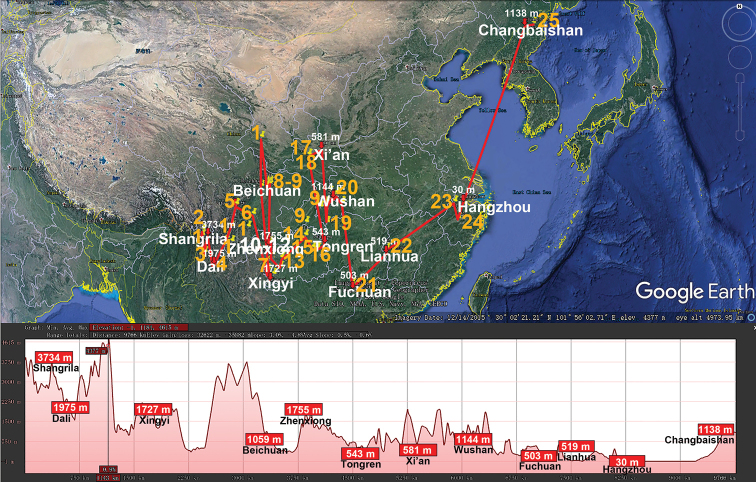
Distribution of the family Polydesmidae, genus *Epanerchodus* in mainland China. Red lines show the transect Shangrila – Dali – Xingyi – Beichuan – Zhengxiong – Tongren – Xi’an – Wushan – Fuchuan – Lianhua – Hangzhou – Changbaishan, along which the elevations are crudely indicated below. **1***E. potanini***2***E. typicus***3***E. fuscus***4***E. yunnanensis***5***E. belousovi***6***E. schawalleri***7***E. stylotarseus***8***E. lipsae***9***E. varius***10***E. frater***11***E. soror***12***E. draco***13***E. coniger***14***E. gladiatus***15***E. chutou***16***E. parvus***17***E. jaegeri***18***E. martensi***19***E. tujiaphiulus***20***E. latus***21***E. orientalis***22***E. jiangxiensis***23***E. enrycornutus***24***E. sphaerisetosus***25***E. koreanus*.

**Figure 7.**
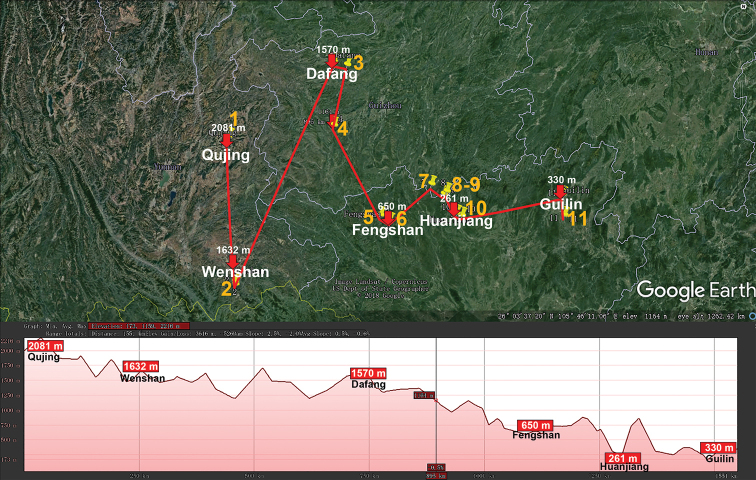
Distribution of the family Polydesmidae, genus *Pacidesmus* in mainland China. Red lines show the transect Qujing – Wenshan – Dafang – Fengshan – Huanjiang – Guilin, along which the elevations are crudely indicated below. **1***P. uncatus***2***P. trilobatus***3***P. martensi***4***P. sinensis***5***P. whitteni***6***P. bifidus***7***P. superdraco***8***P. tiani***9***P. bedosae***10***P. armatus***11***P. trifidus*.

**Figure 8.**
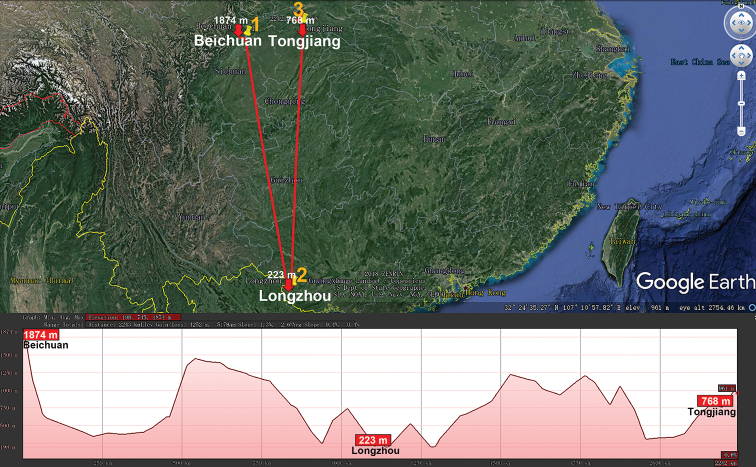
Distribution of the family Polydesmidae, genus *Glenniea* in mainland China. Red lines show the transect Beichuan – Longzhuan – Tongjiang, along which the elevations are crudely indicated below. **1***G. lagredae***2***G. prima***3***G. blanca*.

**Figure 9.**
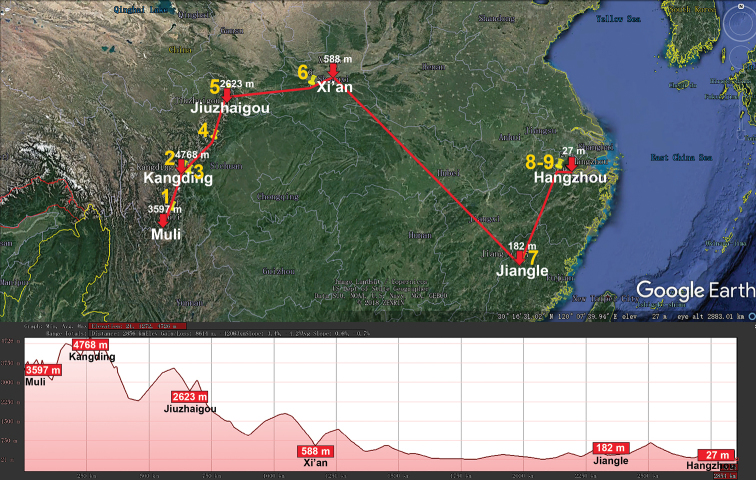
Distribution of the family Xystodesmidae, genus *Riukiaria* in mainland China. Red lines show the transect Muli – Kangding – Jiuzhaigou – Xi’an – Jiangle – Hangzhou, along which the elevations are crudely indicated below. **1***R. belousovi***2***R. kabaki***3***R. spatuliformis***4***R. davidiani***5***R. korolevi***6***R. martensi***7***R. capaca***8***R. chinensis***9***R. tianmu*.

**Figure 10.**
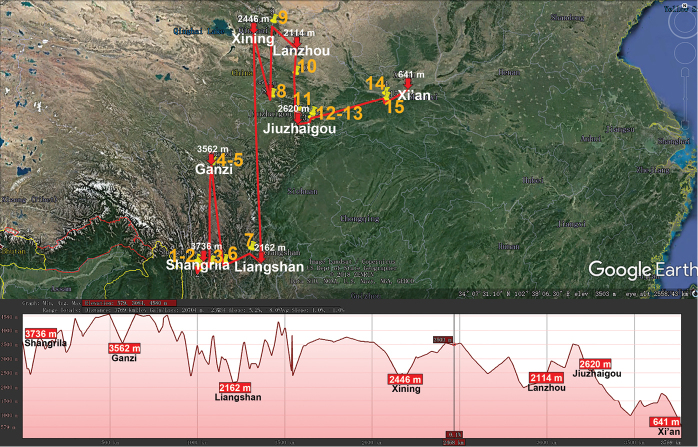
Distribution of the family Paradoxosomatidae, genus *Hedinomorpha* in mainland China. Red lines show the transect Shangrila – Ganzi – Liangshan – Xining – Lanzhou – Jiuzhaigou – Xi’an, along which the elevations are crudely indicated below. **1***H. montana***2***H. yunnanensis***3***H. proxima***4***H. crassiterga***5***H. bifida***6***H. subnigra***7***H. reducta***8***H. martensi***9***H. circofera***10***H. affinis***11***H. nigra***12***H. altiterga***13***H. flavobulbus***14***H. biramipedicula***15***H. jeekeli*; neither *H. circularis* nor *H. hummelii* is mapped because their exact type localities remain unknown.

**Figure 11.**
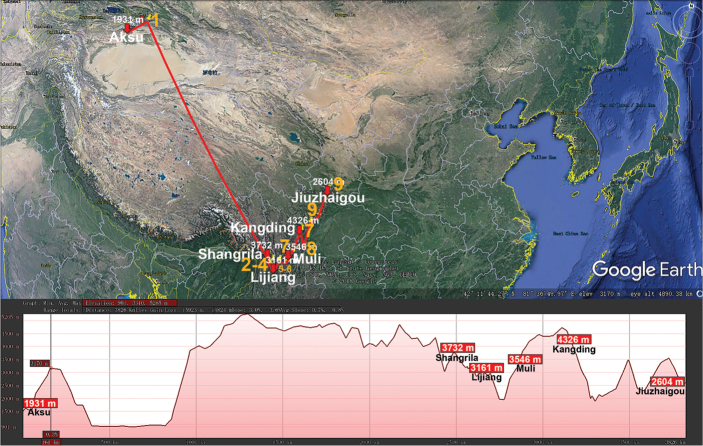
Distribution of the family Paradoxosomatidae, genus *Sigipinius* in mainland China. Red lines show the transect Aksu – Shangrila – Lijiang – Kangding – Jiuzhaigou, along which the elevations are crudely indicated below. **1***S. kabaki***2***S. montana***3***S. spiniger***4***S. dentiger***5***S. campanuliformis***6***S. complex***7***S. simplex***8***S. pinnifer***9***S. grahami*.

**Figure 12.**
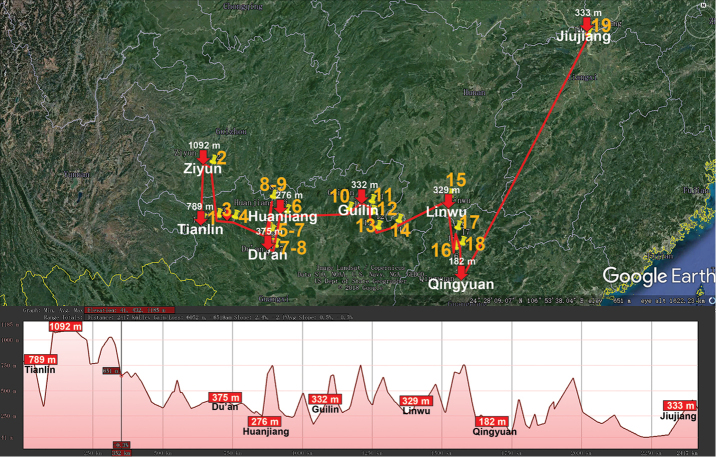
Distribution of the family Paradoxosomatidae, genus *Hylomus* in mainland China. Red lines show the transect Tianlin – Ziyun – Du’an – Huanjiang – Guilin – Linwu – Qingyuan – Jiujiang, along which the elevations are crudely indicated below. **1***H. minutuberculus***2***H. getuhensis***3***H. phasmoides***4***H. variabilis***5***H. parvulus***6***H. scolopendroides***7***H. nodulosus***8***H. scutigeroides***9***H. spinitergus***10***H. lui***11***H. yuani***12***H. cornutus***13***H. lingulatus***14***H. spinissimus***15***H. eupterygotus***16***H. laticollis***17***H. simplipodus***18***H. similis***19***H. draco*; *H. longispinus* is not mapped because its exact type locality remains unknown.

**Figure 13.**
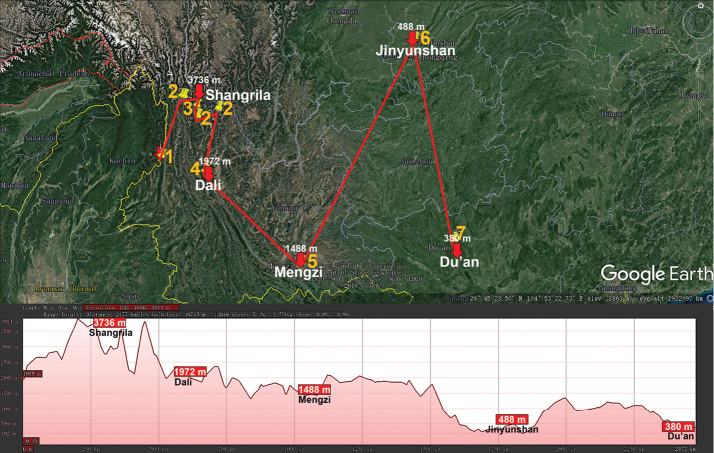
Distribution of the family Paradoxosomatidae, genus *Tylopus* in mainland China. Red lines show the transect Shangrila – Dali – Mengzi – Jinyunshan – Du’an, along which the elevations are crudely indicated below. **1***T. reductus***2***T. kabaki***3***T. similis***4***T. schawalleri***5***T. sinensis***6***T. nigromarginatus***7***T. deharvengi*.

**Figure 14.**
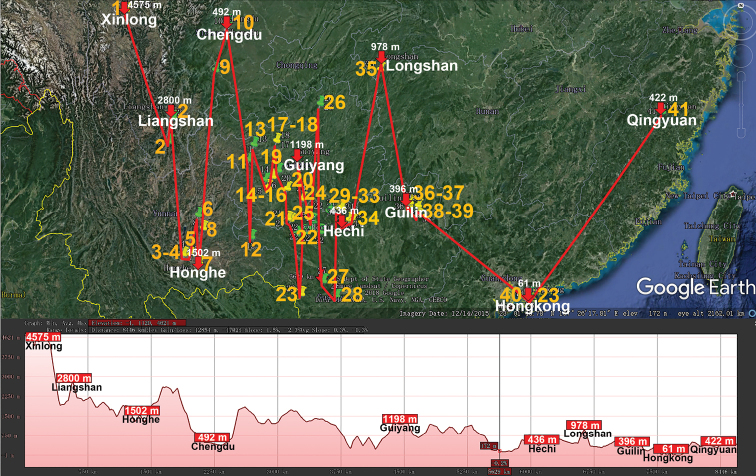
Distribution of the family Cambalopsidae, genus *Glyphiulus* in mainland China. Red lines show the transect Xinlong – Liangshan – Honghe – Chengdu – Guiyang – Hechi – Longshan – Guilin – Hong Kong – Qingyuan, along which the elevations are crudely indicated below. **1***G. basalis***2***G. liangshanensis***3***G. beroni***4***G. paragranulatus***5***G. semigranulatus***6***G. subobliteratus***7***G. subgranulatus***8***G. obliteratus***9***G. latus***10***G. intermedius***11***G. zorzini***12***G. guangnanensis***13***G. foetidus***14***G. sinensis***15***G. pergranulatus***16***G. quadrohamatus***17***G. paracostulifer***18***G. latellai***19***G. obliteratoides***20***G. rayrouchi***21***G. difficilis***22***G. impletus***23***G. granulatus***24***G. basazsi***25***G. calceus***26***G. parobliteratus***27***G. pulcher***28***G. echinoides***29***G. acutus***30***G. mulunensis***31***G. proximus***32***G. tiani***33***G. paramulunensis***34***G. speobius***35***G. deharvengi***36***G. melanoporus***37***G. septentrionalis***38***G. adeloglyphus***39***G. maocun***40***G. formosus***41***G. recticulus*; *G. anophthalmus* and *G. lipsorum* are not mapped because their exact type localities remain unknown, whereas *G. granulatus* is mapped, but it is pantropical.

**Figure 15.**
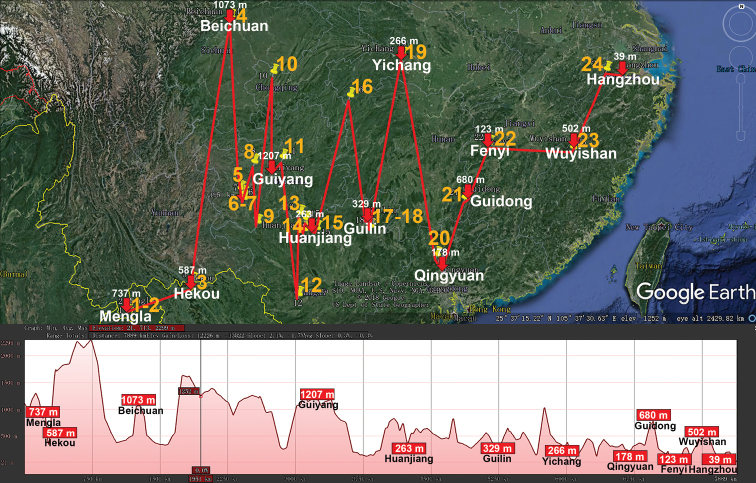
Distribution of the family Haplodesmidae, genus *Eutrichodesmus* in mainland China. Red lines show the transect Mengla – Hekou – Beichuan – Guiyang – Huanjiang – Guilin – Yichang – Qingyuan – Guidong – Fenyi – Wuyishan – Hangzhou, along which the elevations are crudely indicated below. **1***E. dorsiangulatus***2***E. monodentus***3***E. arcicollaris***4***E. triangularis***5***E. tenuis***6***E. latellai***7***E. obliteratus***8***E. incisus***9***E. latus***10***E. soesilae***11***E. triglobius***12***E. distinctus***13***E. trontelji***14***E. planatus***15***E. similis***16***E. sketi***17***E. lipsae***18***E. jianjia***19***E. apicalis***20***E. digitatus***21***E. spinatus***22***E. simplex***23***E. anisodentus***24***E. pectinatidentis*.

Several broken transects have been chosen to grossly reflect the macro relief of mainland China that accompanies the usual mapped distributions (Figs 2–15). The maps and their corresponding transects at the bottom show both horizontal and vertical distributions of all or most species in a number of largely speciose genera from different families and of various origins across China. The species on the maps and along transects are arranged from west to east and/or north to south. The generic level has been chosen as the most suitable to be accepted in historical biogeography (Kryzhanovsky 2002). The above novel approach to a graphic presentation of faunistic data allows us to combine the horizontal and vertical distributions of millipedes in the easiest and most vivid way on the same map. Mapping largely concerns endemic species and only the territory of mainland China.

The colour maps were generated using Google Earth Pro version 7.3.2.5495 and Adobe Photoshop CS6. The final images were processed with Adobe Photoshop CS6.

## Results

The diplopod fauna of continental China at any higher level is basically a mixture of various zoogeographic elements. At the species level, most diplopods encountered in China are not only endemic to the country, but they are also more or less narrowly localised. This holds especially true for cave-dwellers which are usually presumed troglobionts restricted to a single or few adjacent caves. Generally, as the real diversity of millipedes in China has been estimated to amount to no less than 1,000 species (Golovatch 2015a), the list in Table 1, however impressive, seems to represent only ca. 1/3 of the fauna. It is thereby noteworthy that epigean Diplopoda remain especially badly understudied, since much of the collecting and taxonomic exploration efforts still focus on cavernicoles (Golovatch 2015a).

**Table 1.** The millipede fauna of continental China, with data on distributions and basic literature sources.

Taxa	Altitude (m a.s.l.)	Distribution, province/region (main reference/s)
**Order Polyxenida Verhoeff, 1934**		Global
**Family Polyxenidae Lucas, 1840**		Global
Genus *Eudigraphis* Silvestri, 1948		East Asia
1. *Eudigraphis sinensis* Ishii & Liang, 1990	ca. 100	Zhejiang, Hangzhou, Lake Xihu (Ishii and Liang 1990)
Genus *Polyxenus* Latreille, 1802–03		Global
2. *Polyxenus hangzoensis* Ishii & Liang, 1990	ca. 100	Zhejiang, Hangzhou, Lake Xihu (Ishii and Liang 1990)
**Order Glomerida Brandt, 1833**		Holarctic and SE Asia
**Family Glomeridae Leach, 1816**		Holarctic and SE Asia
Genus *Hyleoglomeris* Verhoeff, 1910	145–2810	Balkans, Anatolia, Caucasus, Central, E and SE Asia
3. *Hyleoglomeris albicorporis* Zhang & Zhang, 1995	ca. 1660	Yunnan, Baoshan City, Cave Shihua Dong (Zhang and Zhang 1995)
4. *H. aschnae* Makhan, 2010	ca. 730	Chongqing, Beibei, Mt. Jinyunshan (Makhan 2010b)
5. *H. baxian* Liu & Tian, 2015	ca. 145	Guangxi, Du’an County, Chengjiang Town, Cave Baxian Park Dong (Liu and Tian 2015a)
6. *H. bicolor* (Wood, 1865)	210	Hong Kong, Mt. Taimoshan (Golovatch et al. 2006b)
7. *H. curtisulcata* Golovatch, Liu & Geoffroy, 2012	420	Guangxi, Huanjiang County, Mulun, Cave Gang Lai Dong (Golovatch et al. 2012b)
8. *H. emarginata* Golovatch, 1981	310	Jiangsu, Nanjing City, Mt. Zijinshan (Golovatch 1981, Golovatch et al. 2006b)
9. *H. eusulcata* Golovatch, Geoffroy & Mauriès, 2006	ca. 410	Guizhou, Libo County, caves Latai Dong and Shuijiang Dong (Golovatch et al. 2006b)
10. *H. generalis* Liu & Tian, 2015	550	Guizhou, Cengong County, Shuiwei Town, Cave Jiangjun Dong (Liu and Tian 2015a)
11. *H. getuhensis* Liu & Tian, 2015	ca. 910	Guizhou, Ziyun County, Getuhe National Geopark, Cave Miaoting Dong (Liu and Tian 2015a)
12. *H. grandis* Liu & Tian, 2015	ca. 280	Guangxi, Dahua County, Qibainong Geopark, Cave Qiaoxu Dong (Liu and Tian 2015a)
13. *H. gudu* Golovatch, Liu & Geoffroy, 2012	1365	Guizhou, Anlong County, Cave Hei Dong (Golovatch et al. 2012b)
14. *H. heshang* Golovatch, Liu & Geoffroy, 2012	ca. 700	Guangxi, Xilin County, Cave Zhoubang Dong (Golovatch et al. 2012b)
15. *H. kunnan* Golovatch, Liu & Geoffroy, 2012	420	Guangxi, Huanjiang County, Mulun, Cave Ganxiao Dong (Golovatch et al. 2012b)
16. *H. lii* Golovatch, Liu & Geoffroy, 2012	190	Guangxi, Fuchuan County, Cave Baifu Dong (Golovatch et al. 2012b)
17. *H. maculata* Golovatch, Geoffroy & Mauriès, 2006	ca. 1315	Yunnan, Mengzi County, Cave Laoshao Dong (Golovatch et al. 2006b)
18. *H. mashanorum* Golovatch, Liu & Geoffroy, 2012	ca. 210	Guangxi, Huanjiang County, Mulun, Cave Mashan Dong (Golovatch et al. 2012b)
19. *H. multistriata* Liu & Tian, 2015	ca. 400	Guizhou, Jiangkou County, Nuxi Town, Cave I Dong (Liu and Tian 2015a)
20. *H. mulunensis* Golovatch, Liu & Geoffroy, 2012	ca. 210	Guangxi, Huanjiang County, Mulun, Cave Xia Dong (Golovatch et al. 2012b)
21. *H. nigu* Golovatch, Liu & Geoffroy, 2012	ca. 1120	Guizhou, Qianxi County, Cave Luo Sai Dong (Golovatch et al. 2012b)
22. *H. qiyi* Golovatch, Liu & Geoffroy, 2012	ca. 210	Guangxi, Huanjiang County, Mulun, Cave MinLi Dong (Golovatch et al. 2012b)
23. *H. reducta* Golovatch, Geoffroy & Mauriès, 2006	ca. 1315	Yunnan, Jianshui County, Cave Yan Dong (Golovatch et al. 2006b)
24. *H. rhinoceros* Liu & Tian, 2015	ca. 1025	Guizhou, Anlong County, Dushan Town, Cave Xiniu Dong (Liu and Tian 2015a)
25. *H. rukouqu* Liu & Wynne, 2019	190	Guangxi, Yangshuo County, Cave Shangshuiyan Dong (Liu and Wynne 2019)
26. *H. sinensis* (Brölemann, 1896)	1540–2810	Sichuan, Kangding County, and Tibet (Golovatch et al. 2006b, Liu and Tian 2015a); New record: Sichuan, W of Ningnan County, 3.3 km WSW of Xiaotiancun village
27. *H. tiani* Golovatch, Liu & Geoffroy, 2012	ca. 300	Hunan, Linwu County, Huatang Town, Cave Long Dong (Golovatch et al. 2012b)
28. *H. variabilis* Liu & Tian, 2015	830	Guizhou, Cengong County, Pingle Town, Cave Wanfuchangcheng Dong (Liu and Tian 2015a)
29. *H. wuse* Golovatch, Liu & Geoffroy, 2012	ca. 425	Guizhou, Maolan County, Cave Dongge Dong (Golovatch et al. 2012b)
30. *H. xia* Golovatch, Liu & Geoffroy, 2012	ca. 300	Hunan, Linwu County, Sanhe Town, Tianhe Village, Cave 1 Dong (Golovatch et al. 2012b)
31. *H. xueju* Golovatch, Liu & Geoffroy, 2012	ca. 140	Guangxi, Du’an County, Cave Yaonan Dong (Golovatch et al. 2012b)
32. *H. xuxiakei* Liu & Wynne, 2019	190	Guangxi, Yangshuo County, Cave Guanshan No. 4 Dong (Liu and Wynne 2019)
33. *H. yinshi* Golovatch, Liu & Geoffroy, 2012	1205	Guizhou, Kaiyang County, Cave Xianyan Dong (Golovatch et al. 2012b)
34. *H. youhao* Golovatch, Liu & Geoffroy, 2012	ca. 300	Hunan, Linwu County, Sanhe Town, near Changshali Village, Cave 2 Dong (Golovatch et al. 2012b)
**Order Sphaerotheriida Brandt, 1833**		S and E Africa, Madagascar, Seychelles, Sri Lanka, S India, Himalayas, E China, SE Asia, Australia, New Zealand
**Family Zephroniidae Gray, in Jones, 1843**		Seychelles, Himalayas, E China, SE Asia, Sumatra, Java, Borneo, Sulawesi, Philippines
Genus *Prionobelum* Verhoeff, 1924	10–1500	Vietnam, E China
35. *Prionobelum hainani* (Gressitt, 1941)	375	Hainan, Tai-Pin-ts’uen (Dwa Bi), foot of Mt. Loi Mother (Mauriès 2001)
36. *P. joliveti* Mauriès, 2001	145	Hainan, W of Danzhou (Mauriès 2001)
37. *P. maculosum* (Attems, 1935)	10	Fujian, Fuzhou City (Attems 1935, Mauriès 2001, Wesener 2016)
38. *P. majorinum* (Zhang & Li, 1982)	1200	Hainan, Mt. Diaoluoshan (Zhang and Li 1982c, Mauriès 2001)
39. *P. multidentata* (Wang & Zhang, 1993)	1500	Fujian, Jiangle County, Mt. Longqi (Wang and Zhang 1993b, Wesener 2016)
Genus *Zephronia* Gray, 1832		Himalayas, Myanmar, E China, Indochina, Thailand, Malaysia
40. *Zephronia profuga* Attems, 1936	?	Hong Kong (Attems 1936, Wesener 2016)
**Order Platydesmida Cook, 1895**		Mediterranean, Near East, E and SE Asia, Nearctic, Central America
**Family Andrognathidae Cope, 1869**		E and SE Asia, USA, Mexico
Genus *Brachycybe* Wood, 1964		E and SE Asia, USA
41. *Brachycybe cooki* (Loomis, 1942)	ca. 1090	Jiangxi, S of Jiujiang, Lushan City, Guling Town (Loomis 1942, Shelley et al. 2005)
**Order Polyzoniida Cook, 1895**		Global
**Family Polyzoniidae Gervais, 1844**		Holarctic
Genus *Angarozonium* Shelley, 1997		N Asia
42. *Angarozonium amurense* (Gerstfeldt, 1859)	100–1800	Heilongjiang, mouth of Songari River; also Siberia and Mongolia (Mikhaljova 2017)
**Order Julida Brandt, 1833**		Holarctic, E and SE Asia
**Family Julidae Leach, 1814**		Holarctic, E and SE Asia
Genus *Anaulaciulus* Pocock, 1895	10–3350	Himalaya and E Asia
43. *Anaulaciulus enghoffi* Korsós, 2001	2700	Gansu, Karyn Valley, S wall of Latshi-san Pass (Korsós 2001)
44. *A. otigonopus* Zhang, 1993	ca. 200	Hunan, Changsha City, Mt. Yuelushan (Zhang 1993a)
45. *A. paludicola* (Pocock,1895)	10	Zhejiang, 25 mi of Ningo (Ningbo), Lake Wo-Lee (Causey 1966)
46. *A. tibetanus* Korsós, 2001	2700–3350	Tibet, Dü Chu Valley; Assam, India, 11,000 feet (Korsós 2001)
47. *A. tonginus* (Karsch, 1881)	?	Hong Kong; Taiwan; ? Hunan (Korsós 1994)
48. *A. vallicola* (Pocock, 1895)	?	Zhejiang, 60 mi inland from Sam-moon Bay, Da-zeh Valley (Causey 1966)
Genus *Nepalmatoiulus* Mauriès, 1983	275–3650	Himalaya, E and SE Asia
49. *Nepalmatoiulus brachymeritus* Enghoff, 1987	2810	Sichuan, Kangding (Enghoff 1987b)
50. *N. eulobos* Enghoff, 1987	320	Guangdong, Meizhou City, Mt. Qingliangshan (Enghoff 1987b)
51. *N. fraterdraconis* Enghoff, 1987	ca. 1045	Jiangxi, Jiujiang City, Mt. Lushan, road to Guling (Enghoff 1987b)
52. *N. polyakis* Enghoff, 1987	ca. 275	Sichuan, Suining City (Enghoff 1987b)
53. *N. rhaphimeritus* Enghoff, 1987	2810	Sichuan, Kangding City (Enghoff 1987b)
54. *N. tibetanus* Enghoff, 1987	2750–3650	SE Tibet, Do-Chu Valley, Pasho Distr., near Rombe Gompa (Enghoff 1987b)
55. *N. yunnanensis* Enghoff, 1987	?	Yunnan (Enghoff 1987b)
Genus *Pacifiiulus* Mikhaljova, 1982		Siberia
56. *Pacifiiulus amurensis* (Gerstfeldt, 1859)	100–2500	Heilongjiang, between mouths of Ussuri and Garyn rivers; also Siberia and the Russian Far East (Mikhaljova 2017)
**Family Mongoliulidae Pocock, 1903**		E Asia
Genus *Skleroprotopus* Attems, 1901	125–1190	E Asia
57. *Skleroprotopus confucius* Attems, 1901	ca. 490	Hebei, Zhangjiakou City (Attems 1901)
58. *S. laticoxalis* Takakuwa, 1942	395	Liaoning, Shenyang City (Takakuwa 1942)
59. *S. membramipedalis* Zhang, 1985	ca. 125–150	Beijing, Fangshan, caves Shihua and Yunshui (Zhang 1985a, Vagalinski et al. 2018)
60. *S. serratus* Takakuwa & Takashima, 1949	ca. 1190	Shanxi, Yantou village (Takakuwa and Takashima 1949)
**Family Nemasomatidae Bollman, 1893**		Holarctic
Genus *Orinisobates* Lohmander, 1933		Holarctic E of Ural Mountains
61. *Orinisobates gracilis* (Verhoeff, 1934)	?	Xinjiang, Urumqi, Mt. Tianshan (Verhoeff 1934, Enghoff 1985)
Genus *Sinostemmiulus* Chamberlin & Wang, 1953		China
62. *Sinostemmiulus simplicior* Chamberlin & Wang, 1953	?	Zhejiang, Chenghsien (Cheng County?) (Chamberlin and Wang 1953, Hoffman 1966)
**Family Parajulidae Bollman, 1893**		Nearctic and E Asia
Genus *Karteroiulus* Attems, 1909		Nearctic and E Asia
63. *Karteroiulus niger* Attems, 1909	?	Jiangxi, Tai-an-Long (Enghoff 1987a)
**Order Spirobolida Cook, 1895**		Pantropical
**Family Spirobolidae Bollman, 1893**		E Asia
Genus *Spirobolus* Brandt, 1833	305–3350	E Asia
64. *Spirobolus bungii* Brandt, 1833	?	North of Beijing (Keeton 1960)
65. *S. cincinnalis* Wang & Zhang, 1993	1500	Fujian, Jiangle County, Mt. Longqi (Wang and Zhang 1993b)
66. *S. grahami* Keeton, 1960	ca. 305–3350	Sichuan, Suifu; S of Suifu on the Yunnan border; Mupin; near Yueh-Shi, Granham; Mt. Omeishan; Kweichow: Shih Men Kan (Keeton, 1960); Hubei, Jianshi County (Wang and Zhang 1993b)
67. *S. umbobrochus* Keeton, 1960	ca. 915	Sichuan, Yongshien; Kueichow, Shih Men Kan (Keeton 1960)
68. *S. walkeri* Pocock, 1895	ca. 150–760	Zhejiang, Chusan Island, “Da-laen-Saen”, 30 mi SW of Ningpo (Keeton 1960)
**Order Spirostreptida Brandt, 1833**		Pantropical
**Family Cambalopsidae Cook, 1895**		Himalaya, E and SE Asia, Java, Borneo
Genus *Glyphiulus* Gervais, 1847	105–4150	E and SE Asia, Java, Borneo
69. *Glyphiulus acutus* Golovatch, Geoffroy, Mauriès & VandenSpiegel, 2011	ca. 210	Guangxi, Huanjiang County, Mulun, caves Ganglai Dong and Huobayun Dong (Golovatch et al. 2011a)
70. *G. adeloglyphus* Zhang & Li, 1982	ca. 120	Guangxi, Yangshuo County, Xingping Town (Zhang and Li 1982b)
71. *G. anophthalmus* (Loksa, 1960)	ca. 105	Guangxi (Loksa 1960)
72. *G. balazsi* (Loksa, 1960)	ca. 990 or 835	Guizhou, Luodian County or Longping Town (Loksa 1960)
73. *G. basalis* Golovatch, Geoffroy, Mauriès & VandenSpiegel, 2007	ca. 4150	Sichuan, Xinlong County, Cave Ganchuan Dong (Golovatch et al. 2007a)
74. *G. beroni* Golovatch, Geoffroy, Mauriès & VandenSpiegel, 2007	ca. 1315	Yunnan, Jianshui County, Cave Baguo Dong; and Tonghai County, Cave Xianren Dong (Golovatch et al. 2007a)
75. *G. calceus* Jiang, Guo, Chen & Xie, 2018	900	Guangxi, Tian’e County, Bala Town, Madong village, Hanyaotun, Cave Xianren Dong (Jiang et al. 2018)
76. *G. deharvengi* Golovatch, Geoffroy, Mauriès & VandenSpiegel, 2007	730	Hunan, Longshan County, Huoyan Village, Cave Feihu Dong, Cave Baiyan Dong, Cave Remi Dong (Golovatch et al. 2007a)
77. *G. difficilis* Golovatch, Geoffroy, Mauriès & VandenSpiegel, 2011	ca. 925	Guangxi, Leye County, Yachang Town, Huaping, Cave She Dong and Cave Xiayan Dong (Golovatch et al. 2011b)
78. *G. echinoides* Golovatch, Geoffroy, Mauriès & VandenSpiegel, 2011	ca. 270	Guangxi, Fushui County, Bapen village, Cave II Dong (Golovatch et al. 2011b)
79. *G. foetidus* Jiang, Guo, Chen & Xie, 2018	690–820	Guangxi, Xilin County, Zhoubang village, Cave Zhoubang Dong; Yunnan, Guangnan County, Bamei Town, Ake village, Cave Miaopu Dong (Jiang et al. 2018)
80. *G. formosus* (Pocock, 1895)	ca. 135	Hong Kong (Pocock 1895)
81. *G. granulatus* Gervais, 1847	135–440	Pantropical; Guangxi, Longzhou; Hong Kong; Taiwan (Golovatch et al. 2007a)
82. *G. guangnanensis* Jiang, Guo, Chen & Xie, 2018	690	Yunnan, Guangnan County, Bamei Town, Ake village, Cave Miaopu Dong (Jiang et al. 2018)
83. *G. impletus* Jiang, Guo, Chen & Xie, 2018	320–830	Guangxi, Lingyun County, сaves (Jiang et al. 2018)
84. *G. intermedius* Golovatch, Geoffroy, Mauriès & VandenSpiegel, 2007	ca. 485	Sichuan, Chengdu, Cave Huanlong Dong (Golovatch et al. 2007b)
85. *G. latellai* Golovatch, Geoffroy, Mauriès & VandenSpiegel, 2007	ca. 1495	Guizhou, Qianxi County, Honglin village, Cave Hangtu Dong, Cave Xiao Dong, Cave Xixiang Dong, Cave Dayan Dong, Cave Tiaoshui Dong, Cave Ludiaoai Dong, Cave Shuhuayan Dong, Cave Shuiluo Dong (Golovatch et al. 2007a)
86. *G. latus* Jiang, Lv, Guo & Chen, 2017	ca. 410	Sichuan, Leshan City, Muchuan County, Cave Longgong Dong (Jiang et al. 2017)
87. *G. liangshanensis* Jiang, Lv, Guo & Chen, 2017	ca. 470–1155	Sichuan, Liangshan Yi Autonomous Prefecture, Xichang City, Xixi, Xianren Cave; Miyi County, Baima Town, Cave Zhuanxulong Dong (Jiang et al. 2017)
88. *G. lipsorum* Mauriès & Nguyen Duy-Jacquemin, 1997	ca. 430	Hubei, cave (Mauriès and Nguyen Duy-Jacquemin 1997)
89. *G. maocun* Liu & Wynne, 2019	180	Guangxi, Lingchuan County, Maocun Village, Cave Liangfeng Dong (Liu and Wynne 2019)
90. *G. melanoporus* Mauriès & Nguyen Duy-Jacquemin, 1997	ca. 180	Guangxi, near Guilin, cave (Mauriès and Nguyen Duy-Jacquemin 1997); Xiufeng District, Cave Maomaotou (Liu and Wynne 2019)
91. *G. mulunensis* Golovatch, Geoffroy, Mauriès & VandenSpiegel, 2011	ca. 270	Guangxi, Huanjiang County, Mulun, caves Mashan Dong and Ganglai II Dong (Golovatch et al. 2011a)
92. *G. obliteratoides* Golovatch, Geoffroy, Mauriès & VandenSpiegel, 2007	1400	Guizhou, Anshun City, Liangshuijing, Cave Tianxian Dong (Golovatch et al. 2007b)
93. *G. obliteratus* Golovatch, Geoffroy, Mauriès & VandenSpiegel, 2007	ca. 1315	Yunnan, Mile County, caves Bailong Dong and Houshan Dong (Golovatch et al. 2007b)
94. *G. paracostulifer* Golovatch, Geoffroy, Mauriès & VandenSpiegel, 2007	ca. 1495	Guizhou, Qianxi County, Honglin Town, Cave Laohu Dong (Golovatch et al. 2007b)
95. *G. paragranulatus* Golovatch, Geoffroy, Mauriès & VandenSpiegel, 2007	ca. 1315	Yunnan, Jianshui County, Cave Yan Dong (Golovatch et al. 2007a)
96. *G. paramulunensis* Golovatch, Geoffroy, Mauriès & VandenSpiegel, 2011	211	Guangxi, Huanjiang County, Mulun, caves Shui Dong and Xialan Dong (Golovatch et al. 2011a)
97. *G. parobliteratus* Golovatch, Geoffroy, Mauriès & VandenSpiegel, 2007	ca. 725–860	Guizhou, Suiyang County, Wenquan Town, Shuanghe, Cave Dafeng Dong (Golovatch et al. 2007b)
98. *G. pergranulatus* Golovatch, Geoffroy, Mauriès & VandenSpiegel, 2007	ca. 1065	Guizhou, Guanling County, Huajiang, Cave Da Dong and Cave Anjiada Dong (Golovatch et al. 2007a)
99. *G. proximus* Golovatch, Geoffroy, Mauriès & VandenSpiegel, 2011	ca. 210	Guangxi, Huanjiang County, Mulun, caves Ganxiao Dong and Dongtu Dong (Golovatch et al. 2011a)
100. *G. pulcher* Loksa, 1960	ca. 105	Guangxi, Daxin County, Fulong Town, a cave (Loksa 1960, Jiang et al. 2018)
101. *G. quadrohamatus* Chen & Meng, 1991	ca. 1110	Guizhou, Zhenning County, several caves (Chen and Meng 1991)
102. *G. rayrouchi* Mauriès & Nguyen Duy-Jacquemin, 1997	ca. 390	Guizhou, Maguan, Cave Heiyan Dong (Mauriès and Nguyen Duy-Jacquemin 1997)
103. *G. recticullus* Zhang & Li, 1982	ca. 325	Zhejiang, Qingyuan County (Zhang and Li 1982b)
104. *G. semigranulatus* Golovatch, Geoffroy, Mauriès & VandenSpiegel, 2007	ca. 1315	Yunnan, Mile County, Cave Bailong Dong; Jianshui County, Cave Yanzi Dong (Golovatch et al. 2007a)
105. *G. septentrionalis* Murakami, 1975	ca. 170	Guangxi, Guilin; Japan, Ryukyus, Okinawa Island (Golovatch et al. 2007a)
106. *G. sinensis* (Meng & Zhang, 1993)	ca. 1065	Guizhou, Guanling County, cave (Meng and Zhang 1993)
107. *G. speobius* Golovatch, Geoffroy, Mauriès & VandenSpiegel, 2011	ca. 310	Guangxi, Huanjiang County, caves Xialan Dong and Shenlong Dong (Golovatch et al. 2011a)
108. *G. subgranulatus* Golovatch, Geoffroy, Mauriès & VandenSpiegel, 2007	1313	Yunnan, Mengzi County, cave near footpath to plateau, Pothole No. 2 (Golovatch et al. 2007a)
109. *G. subobliteratus* Golovatch, Geoffroy, Mauriès & VandenSpiegel, 2007	ca. 1685	Yunnan, Shilin County, Cave Zhiyun Dong (Golovatch et al. 2007b)
110. *G. tiani* Golovatch, Geoffroy, Mauriès & VandenSpiegel, 2011	ca. 210	Guangxi, Huanjiang County, Mulun, Cave Dongzai Dong (Golovatch et al. 2011a)
111. *G. zorzini* Mauriès & Nguyen Duy-Jacquemin, 1997	ca. 1105	Guizhou, Shuicheng County, Cave Anjia Yan (Mauriès and Nguyen Duy-Jacquemin 1997)
Genus *Hypocambala* Silvestri, 1897		SE Asia
112. *Hypocambala polytricha* Golovatch, Geoffroy, Mauriès & VandenSpiegel, 2011	ca. 110	Guangxi, Longzhou County, Nonggang, Cave Biji Dong (Golovatch et al. 2011c)
**Family Pericambalidae Silvestri, 1909**		China, Indochina
Genus *Bilingulus* Zhang & Li, 1981		China, Vietnam
113. *Bilingulus sinicus* Zhang & Li, 1981	165	Guangxi, Guilin City, a cave (Zhang and Li 1981a); Yangshuo County, Cave Shangshuiyan; Xiufeng District, Cave Maomaotou; Lingchuan County, Cave Liangfeng Dong (Liu and Wynne 2019)
Genus *Parabilingulus* Zhang & Li, 1981	105–120	China
114. *Parabilingulus aramulus* Zhang & Li, 1981	ca. 120	Guangxi, Yangshuo County, Xingping Town (Zhang and Li 1981a)
115. *P. simplicius* Mauriès & Jacquemin-Nguyen Duy, 1997	ca. 105	Guangxi, Gongcheng County, Cave Heiyan Dong (Mauriès and Nguyen Duy-Jacquemin 1997)
**Family Harpagophoridae Attems, 1909**		Afrotropical, Himalaya, Sri Lanka, S India, E and SE Asia, Sunda Archipelago
Genus *Agaricogonopus* Zhang & Zhang, 1997		China
116. *Agaricogonopus acrotrifoliolatus* Zhang & Zhang, 1997	ca. 870	Yunnan, Xishuangbanna, Mengla County, tropical rainforest (Zhang and Zhang 1997; Pimvichai et al. 2010)
Genus *Junceustreptus* Demange, 1961	650–1895	China
117. *Junceustreptus brevispinus* Zhang, 1985	ca. 650	Yunnan, Xishuangbanna, Mengman (Zhang 1985b; Pimvichai et al. 2010)
118. *J. browningi* Demange, 1962	ca. 1895	Yunnan (Demange 1962; Pimvichai et al. 2010)
119. *J. retrorsus* Hoffman, 1980	ca. 1890	Sichuan, Ning Gyuen Nfu (Hoffman 1980; Pimvichai et al. 2010)
Genus *Prominulostreptus* Pimvichai, Enghoff & Panha, 2010	?	China
120. *Prominulostreptus prominulus* (Demange, 1962)	?	Yunnan, Lou-Fou-Tsouen (Ing-Ka-Tsoue) (Demange 1962; Pimvichai et al. 2010)
Genus *Uriunceustreptus* Zhang & Chang, 1990	650–1750	China, Vietnam
121. *Uriunceustreptus afemorispinus* Zhang & Chang, 1990	ca. 1750	Yunnan, Gejiu City (Zhang and Chang 1990; Pimvichai et al. 2010)
122. *U. bilamellatus* Zhang, 1997	ca. 650	Sichuan (now Chongqing), Youyang County (Zhang et al. 1997)
**Order Chordeumatida Pocock, 1894**		Mostly Holarctic, but also Central and SW South America, Madagascar, Sri Lanka, S India, E and SE Asia, Sunda Archipelago, Philippines, New Guinea, Australia, New Zealand
**Family Guizhousomatidae Mauriès, 2005**		China
Genus *Guizhousoma* Mauriès, 2005	ca. 1495	China
123. *Guizhousoma latellai* Mauriès, 2005	ca. 1495	Guizhou, Qianxi County, Honglin, caves Changtu Dong, Shujiayan, Luosai Dong, Shuiluo Dong, Tiaoshui Dong; and Dafang County, Cave Hei Dong (Mauriès 2005)
**Family Kashmireumatidae Mauriès, 1982**		Himalaya, E and SE Asia
Genus *Lipseuma* Golovatch, Geoffroy & Mauriès, 2006	435–1405	China
124. *Lipseuma bernardi* Golovatch, Geoffroy & Mauriès, 2006	ca. 435	Sichuan, Xinlong County, Three Eyes Cave (Golovatch et al. 2006a, 2007)
125. *L. josianae* Golovatch Geoffroy & Mauriès, 2006	1405	Hubei, Banqiao Town, Cave ChuanDongZi (Golovatch et al. 2006a)
Genus *Vieteuma* Golovatch, 1984	2100–2300	China, Vietnam
126. *Vieteuma hubeiense* Mauriès & Nguyen Duy-Jacquemin, 1997	ca. 2130	Hubei, Shennongjia, Yanziya, Cave Yanzi Dong (Mauriès and Nguyen Duy-Jacquemin 1997)
127. *V. longi* Shear, 2002	2100–2300	Yunnan, Baoshan City, Mt. Gaoligongshan, Nankang, 36 air km SE of Tengchong; and LuoshuiDong, 28 air km SE of Teng Chong (Shear 2002)
**Family Megalotylidae Golovatch, 1978**		Himalaya, Myanmar, E and SE Asia
Genus *Nepalella* Shear, 1979	750–4530	Himalaya, Myanmar, E and SE Asia
128. *Nepalella caeca* Shear, 1999	1795	Guizhou, Shuicheng County, Cave Anjia Yan (Shear 1999, Liu, Wesener et al. 2017c)
129. *N. grandis* Golovatch, Geoffroy & Mauriès, 2006	ca. 1670	Yunnan, Zhenxiong County, Cave Baiyin Dong (Golovatch et al. 2006a)
130. *N. grandoides* Golovatch, Geoffroy & Mauriès, 2006	ca. 750	Sichuan, Beichuan County, caves Yuan Dong and Black Wind Dong (Golovatch et al. 2006a, Liu, Wesener et al. 2017d)
131. *N. griswoldi* Shear, 2002	2100–2300	Yunnan, Baoshan City, Mt. Gaoligongshan, Luoshuidong, 28 air km of Tengcheng (Shear 2002)
132. *N. jinfoshan* Liu, in Liu, Wesener et al., 2017 2017	1500–2100	Chongqing, Jinfoshan, Cave Houshan Dong; Cave Lingguan Dong (Liu, Wesener et al. 2017d)
133. *N. kavanaughi* Shear, 2002	2500	Yunnan, Nujiang, Pianma, native forest on Mt. Gaoligongshan (Shear 2002)
134. *N. lobata* Liu, in Liu, Wesener et al., 2017	1000	Sichuan, Mianyang City, Beichuan County, Cave Liangshui Dong (Liu, Wesener et al. 2017d)
135. *N. magna* Shear, 2002	2300	Yunnan, Baoshan City, Mt. Gaoligongshan, Luoshuidong, 28 air-km of Tengchong (Shear 2002)
136. *N. marmorata* Golovatch, Geoffroy & Mauriès, 2006	ca. 4350	Sichuan, Xinlong County, caves Snake Mouth Dong and Three Eyes Dong (Golovatch et al. 2006a, 2007)
137. *N. pianma* Shear, 2002	2500	Yunnan, Nujiang, Pianma, Mt. Gaoligongshan, native forest (Shear 2002)
138. *N. troglodytes* Liu, in Liu, Wesener et al., 2017	1200–1300	Guizhou, Guiyang City, Xifeng County, Hejiadong village, Cave Hejia Dong; same County, Mushan village, Cave Zhangkou Dong; Guizhou, Qiannan, Longli County, Cave Feilong Dong; Guizhou, Qiannan, Fuquan County, Cave Sanlou Dong (Liu, Wesener et al. 2017d)
139. *N. wangi* Liu, in Liu, Wesener et al., 2017	1300	Chongqing, Wulong County, Huangying Town, Qimenxia, Cave I Dong (Liu, Wesener et al. 2017d)
**Order Callipodida Pocock, 1894**		Holarctic, E and SE Asia
**Family Caspiopetalidae Lohmander, 1931**		Central Asia and China
Genus *Bollmania* Silvestri, 1896		Central Asia and China
140. *Bollmania beroni* Stoev & Enghoff, 2005	ca. 1315	Yunnan, Jianshui County, Cave Yan Dong (Stoev and Enghoff 2005)
**Family Paracortinidae Wang & Zhang, 1993**		China and Vietnam
Genus *Angulifemur* Zhang, 1997	1315	China
141. *Angulifemur tridigitis* Zhang, 1997	ca. 1315	Yunnan, Mengzi City, Cave Niupeng-yanzi Dong (Zhang 1997)
142. *A. unidigitis* Zhang, 1997	ca. 1315	Yunnan, Mengzi City, caves Longbaopo Dong and Laoxiao Dong (Zhang 1997)
Genus *Paracortina* Wang & Zhang, 1993	865–3300	China and N Vietnam
143. *Paracortina carinata* Wang & Zhang, 1993	3300	Yunnan, Shangrila (= Zhongdian) County (Wang and Zhang 1993a)
144. *P. chinensis* Stoev & Geoffroy, 2004	ca. 1670	Yunnan, Zhenxiong County, caves Ke Ma Dong, Da Hei Dong and Liao Jun Dong (Stoev and Geoffroy 2004)
145. *P. leptoclada* Wang & Zhang, 1993	3300	Yunnan, Shangrila (= Zhongdian) County (Wang and Zhang 1993a)
146. *P. serrata* Wang & Zhang, 1993	ca. 1845	Yunnan, Deqin County (Wang and Zhang 1993a)
147. *P. stimula* Wang & Zhang, 1993	3300	Yunnan, Shangrila (= Zhongdian) County (Wang and Zhang 1993a)
148. *P. thallina* Wang & Zhang, 1993	3300	Yunnan, Shangrila (= Zhongdian) County; Sichuan, Batang County (Wang and Zhang 1993a)
149. *P. viriosa* Wang & Zhang, 1993	3300	Yunnan, Shangrila (= Zhongdian) County (Wang and Zhang 1993a); Tibet, Mangkang County (Stoev et al. 2008)
150. *P. voluta* Wang & Zhang, 1993	ca. 2690	Sichuan, Yajiang County (Wang and Zhang 1993a)
151. *P. yinae* Liu & Tian, 2015	ca. 865	Guangxi, Baise City, Longlin County, Tianshengqiao Town, Yanchang village, Cave I (Liu and Tian 2015c)
152. *P. zhangi* Liu & Tian, 2015	ca. 965	Guizhou, Qianxinan Autonomous Prefecture, Ceheng County, Rongdu village, Cave Qiaoxia Dong (Liu and Tian 2015c)
**Family Sinocallipodidae Zhang, 1993**		China and Indochina
Genus *Sinocallipus* Zhang, 1993	1860	China, Laos and Vietnam
153. *Sinocallipus simplopodicus* Zhang, 1993	1860	Yunnan, Hehou City, Cave Xiao Dong (Zhang 1993b)
**Order Polydesmida Pocock, 1887**		Global
**Family Cryptodesmidae Karsch, 1880**		Pantropical
Genus *Trichopeltis* Pocock, 1894	165–1890	Himalaya, E and SE Asia, Malaysia, Sunda Archipelago
154. *Trichopeltis bellus* Liu, Golovatch & Tian, 2017	1530	Yunnan, Qujing City, Luoping County, Machang village, Cave Shuiyuan Dong (Liu et al. 2017a)
155. *T. intricatus* Liu, Golovatch & Tian, 2017	1890	Yunnan, Kunming City, Shilin County, Guishan Town, Cave Haiyi I Dong (Liu et al. 2017a)
156. *T. latellai* Golovatch, Geoffroy, Mauriès & VandenSpiegel, 2010	ca. 1495	Guizhou, Qianxi County, Honglin Town, caves Tiaoshui Dong and Changtu Dong (Golovatch et al. 2010c)
157. *T. liangfengdong* Liu & Wynne, 2019	180	Guangxi, Lingchuan County, Cave Liangfeng Dong (Liu and Wynne 2019)
158. *T. reflexus* Liu, Golovatch & Tian, 2017	ca. 165	Hunan, Chenzhou City, Linwu County, Xianghualing Town, II Dong Cave (Liu et al. 2017a)
**Family Haplodesmidae Cook, 1895**		Himalaya, Myanmar, E and SE Asia, Malaysia, Sunda Archipelago, New Guinea, Melanesia, Australia
Genus *Doratodesmus* Cook, 1895		Sunda Archipelago, China
159. *Doratodesmus grandifoliatus* Zhang, in Zhang & Wang, 1993	ca. 1315	Yunnan, Mengzi County, Cave Longbaopo Dong (Zhang and Wang 1993)
Genus *Eutrichodesmus* Silvestri, 1910	65–1495	E and SE Asia, Sunda Archipelago, Melanesia
160. *Eutrichodesmus anisodentus* (Zhang, 1995)	ca. 385	Fujian, Mt. Wuyishan (Zhang 1995b)
161. *E. apicalis* Golovatch, Geoffroy, Mauriès & VandenSpiegel, 2015	ca. 75	Hubei, Yichang, Yichang County, Grotte des Araignées (Golovatch et al. 2015)
162. *E. arcicollaris* Zhang, in Zhang & Wang, 1993	ca. 170	Yunnan, Hekou County, Cave Huayu Dong (Zhang and Wang 1993, Golovatch et al. 2009a, 2009b)
163. *E. digitatus* Liu & Tian, 2013	ca. 65	Guangdong, Qingyuan City, Jintan Town, Cave Mi Dong (Liu and Tian 2013)
164. *E. distinctus* Golovatch, Geoffroy, Mauriès & VandenSpiegel, 2009	ca. 105	Guangxi, Fusui County, Bapen, Cave 4 Dong (Golovatch et al. 2009b)
165. *E. dorsiangulatus* (Zhang, in Zhang & Wang, 1993)	ca. 635	Yunnan, Mengla County, Cave Baoniujiao Dong (Zhang and Wang 1993, Golovatch et al. 2009a, 2009b)
166. *E. incisus* Golovatch, Geoffroy, Mauriès & VandenSpiegel, 2009	ca. 1495	Guizhou, Qianxi County, Honglin, caves Tiaoshui Dong, Cave Liaojingling Dong, Jiayan Dong, Dakong Dong and Luosai Dong (Golovatch et al. 2009a)
167. *E. jianjia* Liu & Wynne, 2019	190	Guangxi, Yangshuo County, Cave Guanshan No. 4 (Liu and Wynne 2019)
168. *E. latellai* Golovatch, Geoffroy, Mauriès & VandenSpiegel, 2015	1060	Guizhou, Zhenfeng County, Beipanjiang Town, Cave Shui Chi Dong (Water Pool Cave) (Golovatch et al. 2015)
169. *E. latus* Golovatch, Geoffroy, Mauriès & VandenSpiegel, 2009	ca. 560	Guangxi, Leye County, Yachang Nature Reserve, caves Yanwu Dong, Xiayan Dong, Xiaoshui Dong and She Dong (Golovatch et al. 2009a)
170. *E. lipsae* Golovatch, Geoffroy, Mauriès & VandenSpiegel, 2015	ca. 160	Guangxi, Guilin, Grotte des Squelettes (Golovatch et al. 2015)
171. *E. monodentus* (Zhang, in Zhang & Wang, 1993)	ca. 650	Yunnan, Mengla County, Cave Caiyun Dong (Zhang and Wang 1993, Golovatch et al. 2009a, 2009b)
172. *E. obliteratus* Golovatch, Geoffroy, Mauriès & VandenSpiegel, 2015	ca. 1065	Guizhou, Guanling County, Huajiang Town, Cave Huashiban Dong (Slippery Cave) (Golovatch et al. 2015)
173. *E. pectinatidentis* (Zhang, 1995)	ca. 1010	Zhejiang, Lin’an County, Mt. Tianmu (Zhang 1995a)
174. *E. planatus* Liu & Tian, 2013	ca. 550	Guangxi, Hechi City, Liujia Town, Cave Zhenzhuyan Dong (Liu and Tian 2013)
175. *E. sketi* Golovatch, Geoffroy, Mauriès & VandenSpiegel, 2015	730	Hunan, Longshan County, Huoyan, Cave Feihu Dong (Golovatch et al. 2015)
176. *E. similis* Golovatch, Geoffroy, Mauriès & VandenSpiegel, 2009	ca. 310–420	Guangxi, Huanjiang County, Mulun Nature Reserve, caves Gui II Dong and Shenlong Dong (Golovatch et al. 2009a)
177. *E. simplex* Liu & Tian, 2013	130	Jiangxi, Fenyi County, Cave Taoyuan Dong (Liu and Tian 2013)
178. *E. soesilae* Makhan, 2010	ca. 735	Chongqing, Beibei, Mt. Jinyunshan (Makhan 2010a)
179. *E. spinatus* Liu & Tian, 2013	ca. 875	Hunan, Guidong County, Sidu Town, Sidu Caves (Liu and Tian 2013)
180. *E. tenuis* Golovatch, Geoffroy, Mauriès & VandenSpiegel, 2015	ca. 1065	Guizhou, Guanling County, Yongning Town, Cave Yun Dong (Cloud Cave) (Golovatch et al. 2015)
181. *E. triangularis* Golovatch, Geoffroy, Mauriès & VandenSpiegel, 2015	ca. 750	Sichuan, Beichuan County, Cave Yan Dong (Golovatch et al. 2015)
182. *E. troglobius* Golovatch, Geoffroy, Mauriès & VandenSpiegel, 2015	1205	Guizhou, Kaiyang County, Cave Xianyan Dong (Golovatch et al. 2015)
183. *E. trontelji* Golovatch, Geoffroy, Mauriès & VandenSpiegel, 2015	ca. 410	Guizhou, Libo County, caves Shui Jiang Dong, Shuipu Da Dong, Shuipa, Latai Dong, Jia Ban and Feng Dong (Golovatch et al. 2015)
**Family Paradoxosomatidae Daday, 1889**		Global except for N America
Genus *Anoplodesmus* Pocock, 1895		S, E and SE Asia
184. *Anoplodesmus chinenis* Golovatch, 2013	1700–2400	Shaanxi, Mt. Taibaishan, southern slopes, above Houshenzi, primary broadleaved forest (Golovatch 2013a)
Genus *Antheromorpha* Jeekel, 1968		E and SE Asia
185. *Antheromorpha rosea* Golovatch, 2013	1200–1700	Yunnan, S of Pianma; Baoshan District, near Hemu, Mt. Gaoligongshan, near Cave Bianfu II Dong (Golovatch 2013a, 2013b); also N Thailand and Laos (Likhitrakarn et al. 2019)
Genus *Belousoviella* Golovatch, 2012		China
186. *Belousoviella kabaki* Golovatch, 2012	3360	Sichuan, SW of Mianning, right tributary of Yalongjiang River canyon (Golovatch 2012)
Genus *Cawjeekelia* Golovatch, 1980	100–2110	E and SE Asia
187. *Cawjeekelia nova* Golovatch, 2011	2110	Chongqing, Dabashan Mt. Range, NE of Heyu, *Betula* forest (Golovatch 2011)
188. *C. pallida* Golovatch, 1996	100–200	Hong Kong, Tai Po Kau Nature Reserve (Golovatch 1996)
189. *C. propria* (Mikhaljova & Korsós, 2003)	500	Jilin, Mt. Changbaishan National Park; also N Korea (Mikhaljova and Korsós 2003, Golovatch 2013a)
Genus *Desmoxytes* Chamberlin, 1923		E and SE Asia
190. *Desmoxytes planata* (Pocock, 1895)	560	Nearly pantropical; Yunnan, Xishuangbanna, Menglun, Tropical Botanical Garden (Srisonchai et al. 2018; Golovatch 2018)
Genus *Enghoffosoma* Golovatch, 1993		E and SE Asia
191. *Enghoffosoma longipes* Golovatch, 2011	3150	Yunnan, NW slope of Mt. Yulongxueshan (Golovatch 2011)
Genus *Gonobelus* Attems, 1936	995–2615	China
192. *Gonobelus belousovi* Golovatch, 2014	995	Sichuan, NE of Shimian, Xiangshuigou River, Tianpingzi (Golovatch 2014a)
193. *G. martensi* Golovatch, 2013	1700–2600	Shaanxi, Mt. Taibaishan (Golovatch 2013a)
194. *G. pentaspinus* Golovatch, 2013	2475	Sichuan, NW of Mianning (Golovatch 2013b)
195. *G. sinensis* Attems, 1936	2615	Yunnan, Mt. Laojunshan, 3.7 km ENE of Segengsheng (Golovatch 2017)
Genus *Hedinomorpha* Verhoeff, 1934	1300–4490	Central Asia and China
196. *Hedinomorpha affinis* Golovatch, 2014	2870	Gansu, Mt. Lianhuashan (Golovatch 2014a)
197. *H. altiterga* Golovatch, 2019	1445	Gansu, WWS of Longnan (Wudu), 2.4 km NW of Zhongzhaixiang (Golovatch 2019a)
198. *H. bifida* Golovatch, 2019	3665	Sichuan, 7.3 km S of Ganzi (Golovatch 2019a)
199. *H. biramipedicula* Zhang & Tang, 1985	ca. 1360	Shaanxi, Qinling, Mt. Taibaishan (Zhang and Tang 1985)
200. *H. circofera* Golovatch, 2013	ca. 2735	Qinghai, Beishan National Park, 120 km N of Xining (Golovatch 2013a)
201. *H. circularis* (Takakuwa & Takashima, 1949)	?	Shanxi, Chinkaiji (Takakuwa and Takashima 1949, Golovatch 2019a)
202. *H. crassiterga* Golovatch, 2019	4490	Sichuan, 16.8 km SSW Ganzi (Golovatch 2019a)
203. *H. flavobulbus* Golovatch, 2019	3650	Gansu, WWS of Longnan (Wudu), Yin Duoguosa & Aounang divide (Golovatch 2019a, 2019b)
204. *H. hummelii* Verhoeff, 1934	?	Gansu, Tan-Chang (Verhoeff 1934)
205. *H. jeekeli* (Golovatch, 2009)	1300–2600	Shaanxi, Foping Nature Reserve, Panda area (Golovatch 2009); Shaanxi, Mt. Taibaishan, S slopes, above Houshenzi, primary and secondary broadleaved forests (Golovatch 2013a)
206. *H. martensi* Golovatch, 2014	3510	Sichuan, Langmusi, remnants of a moist *Abies* forest above town (Golovatch 2014a)
207. *H. montana* Golovatch, 2016	3080–3695	Yunnan, NNE of Weixi City, 8.15 km ESE of Shajiama; N of Weixi City, 2.95 km NW of Xugongqingshang Village; NW of Jianchuan, 4.7 km WNW of Damaidi; Mt. Laojunshan, NE of Liming, 4.2 km S of Muzhengdu (Golovatch 2016b, 2017)
208. *H. nigra* Golovatch, 2013	3530–4000	Sichuan, Jiuzhaigou County, N of Dajisi (Golovatch 2013b)
209. *H. proxima* Golovatch, 2016	3570	Yunnan, Mt. Tianbaoshan between Shangrila and Mt. Habaxueshan, E slope, NW of Bengla (Golovatch 2016b)
210. *H. reducta* Golovatch, 2012	2900	Sichuan, SW of Mianning, Right tributary of Yalongjiang River canyon, ca. 9 km SW of Mofanggou (Golovatch 2012)
211. *H. subnigra* Golovatch, 2013	3910	Yunnan, W of Lake Lugu (Golovatch 2013b)
212. *H. yunnanensis* Golovatch, 2016	3480	Yunnan, NNE of Weixi City, right tributary of Lapugon River, 5.2 km ENE of Jizong (Golovatch 2016b)
Genus *Helicorthomorpha* Attems, 1914		E and SE Asia
213. *Helicorthomorpha holstii* (Pocock, 1895)	340	Widespread in SE Asia; Yunnan; Guangdong, Dinghushan Mt., 86 km W of Guangzhou (Attems 1936, Golovatch 1981)
Genus *Hirtodrepanum* Golovatch, 1994		Himalaya and China
214. *Hirtodrepanum chinense* Golovatch, 2014	1990–2015	Yunnan, Deqin, Dewei Line, E of Aqiku; Mekong Valley, 2 km E of Yezhixiang (Golovatch 2014a, 2019a)
Genus *Hylomus* Cook & Loomis, 1924	ca. 140–910	E and SE Asia
215. *Hylomus cornutus* (Zhang & Li, 1982)	ca. 140	Guangxi, Guilin, Yangshuo (Zhang and Li 1982a)
216. *H. draco* Cook & Loomis, 1924	ca. 400	Jiangxi, Jiujiang City, Mt. Lushan (Cook and Loomis 1924, Srisonchai et al. 2018)
217. *H. eupterygotus* (Golovatch, Li, Liu & Geoffroy, 2012)	ca. 260	Hunan, Linwu County, Tianhe, Cave I Dong and Changshali Cave I Dong (Golovatch et al. 2012a)
218. *H. getuhensis* (Liu, Golovatch & Tian, 2014)	ca. 910	Guizhou, Ziyun County, Getuhe National Geopark, caves Suidao Dong and Taiyang Dong (Liu et al. 2014)
219. *H. laticollis* (Liu, Golovatch & Tian, 2016)	450	Guangdong, Yingde City, Huanghua Town, Yanbei village, Cave Yangyan Dong (Liu et al. 2016)
220. *H. lingulatus* (Liu, Golovatch & Tian, 2014)	ca. 140	Guangxi, Guilin, Pingle County, Cave Chaotianyan (Liu et al. 2014)
221. *H. longispinus* (Loksa, 1960)	?	Guangxi, a cave (no exact locality known) (Loksa 1960)
222. *H. lui* (Golovatch, Li, Liu & Geoffroy, 2012)	ca. 155	Guangxi, Yongfu County, Shangxiao, Cave Dachong Dong (Golovatch et al. 2012a)
223. *H. minutuberculus* (Zhang, 1986)	ca. 295	Guangxi, Tianlin County (Zhang 1986)
224. *H. nodulosus* (Liu, Golovatch & Tian, 2014)	ca. 350	Guangxi, Du’an County, Xia’ao Town, near Xia’ao Middle School, Cave II Dong; same county, Yong’an Town, Yong’an village, Cave I Dong; same town, Anju Village, Cave Suidao Dong; same county, Longwan Town, Qunle village, entrance to Cave I Dong (Liu et al. 2014)
225. *H. parvulus* (Liu, Golovatch & Tian, 2014)	ca. 350	Guangxi, Du’an, Xia’ao (Liu et al. 2014)
226. *H. phasmoides* (Liu, Golovatch & Tian, 2016)	ca. 445	Guangxi, Lingyun County, Jiayou Town, Yangli village, Cave Fengliu Dong (Liu et al. 2016)
227. *H. scolopendroides* (Golovatch, Geoffroy & Mauriès, 2010)	ca. 210–350	Guangxi, Huanjiang County, Dacai Town, Cave Shenlong Dong; Du’an County, Gaoling Town, Jinzhu village, Cave I Dong, Cave II Dong; Xia’ao Town, Cave I Dong (Golovatch et al. 2010b, Liu et al. 2014)
228. *H. scutigeroides* (Golovatch, Geoffroy & Mauriès, 2010)	ca. 310	Guangxi, Huanjiang County, Cave Ganglai Dong, Cave Mashan II Dong, Cave Gonglu Dong, Cave Shui Dong, and Du’an County, Disu Town, Dading village, Cave II Dong, same county, Longwan Town, Nongqu village, Cave I Dong, (Golovatch et al. 2010b, Liu et al. 2014)
229. *H. similis* (Liu, Golovatch & Tian, 2016)	230	Guangdong, Yingde City, Qingkeng Town, Bangjiao village, Cave Bangjiao Dong (Liu et al. 2016)
230. *H. simplipodus* (Liu, Golovatch & Tian, 2016)	140	Guangdong, Qingyuan City, Yangshan County, Chengjia Town, Dabei Village, Cave Kuangzhanyan (Liu et al. 2016)
231. *H. spinissimus* (Golovatch, Li, Liu & Geoffroy, 2012)	190	Guangxi, Fuchuan County, Guanyuan, Cave Guanyuan Dong (Golovatch et al. 2012a)
232. *H. spinitergus* (Liu, Golovatch & Tian, 2016)	ca. 210	Guangxi, Huanjiang County, near Cave Gui DongII, secondary forest (Liu et al. 2016)
233. *H. variabilis* (Liu, Golovatch & Tian, 2016)	500	Guangxi, Fengshan County, numerous caves (Liu et al. 2016)
234. *H. yuani* Liu & Wynne, 2019	180	Guangxi, Lingchuan County, Cave Liangfeng (Liu and Wynne 2019)
Genus *Inversispina* Zhang, 1997	510–4150	China and Taiwan
235. *Inversispina erectispina* Golovatch, 2012	2400–4150	Sichuan, SW of right tributary of Yalongjiang River, canyon; Sichuan, NW of Mianning, broadleaved forest; Sichuan, Jiulong County, SW of Wulaxixiang, broadleaved forest; Yunnan, between Tianbaoshan and Luzilashan, between Shuimofang and Xipazi; Yunnan, N of Lijiang, NW of Baoshanxiang, W of Bengluo village (Golovatch 2012, 2013b, 2016a, 2016b)
236. *I. multispina* Golovatch, 2016	2360	Sichuan, SSE of Shimian, S of Zhuma (Golovatch 2016a)
237. *I. tortiapicalis* Zhang, 1997	510	Hubei, Hefeng Tu jiazu County, Yien (Zhang et al. 1997)
238. *I. trispina* Golovatch, 2013	1050	Sichuan, Mt. Emeishan, Wannian Monastery (Golovatch 2013a)
Genus *Kronopolites* Attems, 1914	35–3600	Himalaya, E and SE Asia
239. *Kronopolites biagrilectus* Hoffman, 1963	35–3600	Jiangxi, 10 mi S of Jiujiang (oHoffman 1963); Sichuan, SSE of Shimian, S of Zhuma; Yunnan, Mt. Laojunshan,NE Liming, 2.5 km SE of Yankuluo; N of Lanping, 10.3 km SW of Hexi; N of Lanping, 11.3 km SW of Hexi; Yunnan, SE of Deqen City, 3.3 km S of Gejiancun; Yunnan, Mt. Laojunshan, NE Liming, 2.5 km SE of Yankuluo; N of Lanping, 10.3 km SW of Hexi; N of Lanping, 11.3 km SW of Hexi (Golovatch 2016a, 2016b, 2017)
240. *K. davidiani* Golovatch, 2014	3365	Sichuan, Wenchuan City, 214 National Road, WSW of Edi (Golovatch 2014a)
241. *K. swinhoei* (Pocock, 1895)	1300–1700	Shaanxi, Mt. Taibaishan (oHoffman 1963); Shaanxi, Panda area, Foping Nature Reserve; Gansu, WWS of Longnan (Wudu), 2.4 km NW of Zhongzhaixiang (Golovatch 2017, 2019a)
Genus *Mandarinopus* Verhoeff, 1934	700–2955	China
242. *Mandarinopus corticinus* (Attems, 1936)	?	Yunnan (Attems 1936, Golovatch 2019a)
243. *M. gracilipes* Verhoeff, 1934	700–2195	Gansu, Baishui Jiang River; WWS of Longnan (Wudu), 3 km W of Jiejiaonuocun, Yin Duoguosa (Verhoeff 1934, Golovatch 2019a)
244. *M. hirsutus* Golovatch, 2019	2315	Yunnan, NW of Lijiang, W of Chang Jiang (= Yangtze) River, NW of Jinzhuang, 2.5 km N of Tuozhi village (Golovatch 2019a)
245. *M. rugosus* (Golovatch, 2013)	2400	Yunnan, N of Lijiang (Golovatch 2013a, 2019a)
246. *M. semirugosus* (Golovatch, 2013)	2955	Sichuan, NW of Mianning (Golovatch 2013b, 2019a)
Genus *Nedyopus* Attems, 1914	170–450	E and SE Asia
247. *Nedyopus beroni* (Golovatch, 1995)	350–450	Jiangsu, Nanjing City, Mt. Zijin (Golovatch 1995)
248. *N. picturatus* (Golovatch, 1995)	ca. 170	Guangxi, Guilin (Golovatch 1995)
Genus *Orthomorpha* Bollman, 1893		E and SE Asia, Sunda Archipelago
249. *Orthomorpha coarctata* (de Saussure, 1860)	ca. 20	Pantropical; Hainan, Sanya (Golovatch 1994)
250. “*Orthomorpha*” endeusa Attems, 1898	?	China (Attems 1898)
Genus *Orthomorphella* Hoffman, 1963		China
251. *Orthomorphella pekuensis* (Karsch, 1881)	ca. 40–165	Hebei, Shanlin, 70 km of Peking (Golovatch 1981); Hunan, Yuanling County, Mumaling (Zhang et al. 1997); New record: Jilin, Changchun City.
Genus *Oxidus* Cook, 1911		E and SE Asia
252. *Oxidus gracilis* C. L. Koch, 1847	200–1300	Subcosmopolitan, anthropochore; near Beijing; Shaanxi, Xi’an City; Guangxi, near Guilin; Sichuan, Maoxian County, NE of Shimian (Golovatch 2013a, 2014a)
Genus *Piccola* Attems, 1953		China and Vietnam
253. *Piccola golovatchi* Liu & Tian, 2015	ca. 840	Guangxi, Baise City, Tianlin County, Langping Town, Cave Shizikou Dadong (Liu and Tian 2015b)
Genus *Polylobosoma* Jeekel, 1980	10–1600	China and Vietnam
254. *Polylobosoma panda* (Golovatch, 2009)	1600	Shaanxi, Foping Nature Reserve, Panda area (Golovatch 2009, 2014a)
255. *P. roseipes* (Pocock, 1895)	10	Zhejiang, Ningpo (Jeekel 1980)
Genus *Sellanucheza* Enghoff, Golovatch & Nguyen, 2004	995–3155	E and SE Asia
256. *Sellanucheza jaegeri* Golovatch, 2013	1300–1700	Shaanxi, Mt. Taibaishan (Golovatch 2013a)
257. *S. tenebra* (Hoffman, 1961)	?	Sichuan, Wushan (Hoffman 1961)
258. *S. typica* Golovatch, 2013	995–3155	Sichuan, Maoxian County, SE of Nanxizhen (Golovatch 2013b); Sichuan, NE of Shimian, Xiangshuigou River, Tianpingzi (Golovatch 2014a)
Genus *Sigipinius* Hoffman, 1961	2810–4195	China
259. *Sigipinius campanuliformis* Golovatch, 2013	3910	Yunnan, W of Lake Lugu, N of Dajisi (Golovatch 2013b)
260. *S. complex* Golovatch, 2013	3780–4120	Sichuan, S of Muli (Golovatch 2013b)
261. *S. dentiger* Golovatch, 2016	3570	Yunnan, Mt. Tianbaoshan between Shangrila and Habaxue Shan, E slope, NW of Bengla (Golovatch 2016b)
262. *S. grahami* Hoffman, 1961	2810–4170	Sichuan, Lixi County, SW of Tonghua; Jiuzhaigou County, N of Dajisi; Maoxian County, SE of Nanxizhen; Lixian, NNW of Xuecheng, Ertaizi; N of Lixian, Mengdonggou & Lianghekou divide, W of Xing Fanweizi; Gansu, WWS of Longnan (Wudu), Yin Duoguosa & Aounang divide; WWS of Longnan (Wudu), Yin Duoguosa & Yaxielu, W of Zhagazu, WWS of Longnan (Wudu), Wushenggou & Line Chaping divide; NNE Zhugqu, Minjiang Bas, 3 km ENE Xiaohuangya, Qinyugou (Golovatch 2013b, 2019a)
263. *S. kabaki* Golovatch, 2013	3330–3550	Xinjiang, Koeksu Basin (Golovatch 2013b)
264. *S. montanus* (Golovatch, 2011)	3710–4090	Yunnan, S of Nixi, near upper timber-line of a humid montane *Abies* forest; WNW of Zhongdian, humid mid-montane *Abies* forest with admixture of broad-leaved hardwood species (Golovatch 2011, 2013b)
265. *S. pinnifer* Golovatch, 2016	3625	Sichuan, SSE of Shimian, S of Zhuma (Golovatch 2016a)
266. *S. simplex* Golovatch, 2013	3915–4195	Sichuan, Jiulong County, SW of Wulaxixiang; Muli County, SW of Wulaxixiang (Golovatch 2013b); Sichuan, Kangding NNE of Yalaxiang, Shuangyanwo (Golovatch 2014a)
267. *S. spiniger* Golovatch, 2014	3690–3960	Yunnan, from Lijiang to Shangrila, 214 National Road, WSW of Edi (Golovatch 2014a)
Genus *Sinomorpha* Golovatch, 2013		China
268. *Sinomorpha setosa* Golovatch, 2013	1050	Sichuan, Mt. Emeishan, Wannian Monastery (Golovatch 2013a)
Genus *Tetracentrosternus* Pocock, 1895		Myanmar, Thailand and Indochina
269. *Tetracentrosternus hoffmani* Golovatch, 2013	1610	Yunnan, Mt. Gaolinggongshan, S of Pianma (Golovatch 2013a)
Genus *Tonkinosoma* Jeekel, 1953	500–1250	China and Vietnam
270. *Tonkinosoma flexipes* Jeekel, 1953	500	Guangxi, Hechi City, Fengshan County, Jinya Town, Hangdong village (Liu and Golovatch 2018a); also N Vietnam (Jeekel 1953)
271. *T. tiani* Liu & Golovatch, 2018	1250	Guizhou, Qianxinan, Anlong County, Sayu Town, Ganhan Dong Cave (Liu and Golovatch 2018a)
Genus *Tylopus* Jeekel, 1968	350–4025	Myanmar, China, Thailand and Indochina
272. *Tylopus deharvengi* Liu & Luo, 2013	350	Guangxi, Du’an County, Xia’ao Town, Cave I Dong (Liu and Luo 2013)
273. *T. kabaki* Golovatch, 2014	3575–4025	Yunnan, Deqen, Tuoxia Highway, Mt. Xiaruolisuzuxiang & Yezhizhen; same province, NW of Lijiang, W of Chang Jiang (Yangtze River), NW of Jinzhuang, 6 km of Tuozhi village; N of Lijiang, W of Maguwa, 4.2 km SE of Shanggaohan village; N of Lijiang, W of Maguwa, 4.4 km ENE of Shanggaohan village; Mekong Valley, ENE of Yezhixiang, 3 km NE of Houqing (Golovatch 2014a, 2019b)
274. *T. nigromarginatus* Golovatch, 2018	835	Chongqing, Mt. Jinyunshan, secondary forest, stump, trees, small cave (Golovatch 2018)
275. *T. reductus* Golovatch, 2013	1600–1800	Yunnan, Mt. Gaoligongshan, S of Pianma (Golovatch 2013a)
276. *T. schawalleri* Golovatch, 2013	2500–2700	Yunnan, Mt. Dincangshang, above Dali (Golovatch 2013a)
277. *T. similis* Golovatch, 2014	1670	Yunnan, from Lijiang to Shangrila, E of Guojie Luocun (Golovatch 2014a)
278. *T. sinensis* Golovatch, 1995	1315	Yunnan, Mengzi County, Cave Hafatiao Dong (Golovatch 1995)
Genus *Wulingina* Zhang, 1997		China
279. *Wulingina macroloba* Zhang, 1997	510	Hubei, Hefeng Tu jiazu County (Zhang 1997)
280. *W. miniloba* Zhang, 1997	510	Hubei, Hefeng Tu jiazu County (Zhang 1997)
Genus *Yuennanina* Attems, 1936	1915–1920	China
281. *Yuennanina aceratogaster* Zhang & Li, 1977	1920	Yunnan, Kunming City (Zhang and Li 1977)
282. *Y. ceratogaster* Attems, 1936	1920	Yunnan, Kunming City (Attems 1936)
283. *Y. petalolobodes* Chang & Zhang, 1989	1915	Yunnan, Kunming, Chenggong County (Chang and Zhang 1989)
**Family Polydesmidae Leach, 1815**		Palaearctic and SE Asia
Genus *Epanerchodus* Attems, 1901	35–3090	Central and E Asia, marginally N Vietnam
284. *Epanerchodus belousovi* Golovatch, 2014	2810	Sichuan, Kangding City (Golovatch 2014c)
285. *E. chutou* Liu & Golovatch, 2018	680	Guizhou, Shiqian County, Cave Feng Dong (Liu and Golovatch 2018b)
286. *E. coniger* Liu & Golovatch, 2018	ca. 1620	Guizhou, Bijie City, Zhijin County, Chengguan Town, Dongshan village, Cave Houshan Dong (Liu and Golovatch 2018b)
287. *E. draco* Geoffroy & Golovatch, 2004	ca. 1670	Yunnan, Zhenxiong County, a cave; Guizhou, Liupanshui City, Shuicheng County, Cave Shendongmigong Dong (Geoffroy and Golovatch 2004, Liu and Golovatch 2018b)
288. *E. eurycornutus* Zhang & Wang, 1992	885	Zhejiang, Mt. Tianmu (Zhang and Wang 1992)
289. *E. frater* Geoffroy & Golovatch, 2004	ca. 1670	Yunnan, Zhenxiong County, Cave Dahei Dong (Geoffroy and Golovatch 2004)
290. *E. fuscus* Golovatch, 2015	ca. 2450	Yunnan, Lanping County (Golovatch 2015b)
291. *E. gladiatus* Liu & Golovatch, 2018	920	Guizhou, Wuchuan County, Huangdu Town, Gaodong village, Cave Yinshi Dong (Liu and Golovatch 2018b)
292. *E. jaegeri* Golovatch, 2014	ca. 2345	Shaanxi, Mt. Taibaishan (Golovatch 2014b)
293. *E. jiangxiensis* Liu & Golovatch, 2018	475	Jiangxi, Lianhua County, Gaotan village, Cave Shuilian Dong (Liu and Golovatch 2018b)
294. *E. koreanus* Verhoeff, 1937	2230	Jilin, Mt. Changbaishan (Golovatch 2014b)
295. *E. latus* Liu & Golovatch, 2018	ca. 1330	Chongqing, Wushan County, Luoping Town, Qinglong village, Cave Qinglong Dong (Liu and Golovatch 2018b)
296. *E. lipsae* Golovatch & Geoffroy, 2014	ca. 750	Sichuan, Beichuan and Jiangyou counties, numerous caves (Golovatch and Geoffroy 2014, Liu and Golovatch 2018b)
297. *E. martensi* Golovatch, 2014	ca. 2345	Shaanxi, Mt. Taibaishan (Golovatch 2014b)
298. *E. orientalis* Attems, 1901	ca. 205	Guangxi, Fuchuan County, Cave Banbianshan Dong (Golovatch et al. 2012c), also Japan and Taiwan
299. *E. parvus* Liu & Golovatch, 2018	830	Guizhou, Cengong County, Pingzhuang Town, Cave Wanfuchangcheng Dong (Liu and Golovatch 2018b)
300. *E. potanini* Golovatch, 1991		Sichuan, Gansu and Yunnan provinces (Golovatch 1991a, 2014b)
301. *E. schawalleri* Golovatch, 2014	ca. 1550	Sichuan, Mt. Emeishan (Golovatch 2014b)
302. *E. soror* Geoffroy & Golovatch, 2004	ca. 1670	Yunnan, Zhenxiong County, caves Hama Dong, Dahei Dong and Xianren Dong (Geoffroy and Golovatch 2004, Liu and Golovatch 2018b)
303. *E. sphaerisetosus* Zhang & Chen, 1983	ca. 35	Zhejiang, 10 mi S of Jinhua City, Gaocun village (Zhang and Chen 1983)
304. *E. stylotarseus* Chen & Zhang, 1990	ca. 1220	Guizhou, Guanling County, several caves (Chen and Zhang 1990, Golovatch et al. 2007, 2012)
305. *E. tujiaphilus* Liu & Golovatch, 2018	730	Hunan, Longshan County, Huoyan village, Cave Tujiamei Dong (Liu and Golovatch 2018b)
306. *E. typicus* Golovatch, 2014	ca. 3030	Yunnan, Deqin County (Golovatch 2014c)
307. *E. varius* (Geoffroy & Golovatch, 2004)	ca. 755–3090	Numerous caves in Hubei, Banqiao Town; and Sichuan, Xinlong and Beichuan counties (Geoffroy and Golovatch 2004, Golovatch et al. 2007, Golovatch and Geoffroy 2014)
308. *E. yunnanensis* Golovatch, 2014	1995	Yunnan, Dali City (Golovatch 2014b)
Genus *Glenniea* Turk, 1945	170–1510	Himalaya and China
309. *Glenniea blanca* Golovatch & Geoffroy, 2014	600	Sichuan, Tongjiang County, Cave Lou Fang Dong (= Grotte de la Maison) (Golovatch and Geoffroy 2014)
310. *G. lagredae* Golovatch & Geoffroy, 2014	1360–1510	Sichuan, Beichuan County, Cave Yuan Dong (= La grotte du Rocher); Sichuan, Huajiaoling County, Cave Zhangjiayankoukeng Dong (Golovatch and Geoffroy 2014)
311. *G. prima* Golovatch, Li, Liu & Geoffroy, 2012	ca. 170	Guangxi, Longzhou County, Shanglong Town, Lenglei Nonggang Forest (Golovatch et al. 2012c, Golovatch and Geoffroy 2014)
Genus *Pacidesmus* Golovatch, 1991	ca. 180–1865	China and N Thailand
312. *Pacidesmus armatus* Golovatch, Geoffroy & Mauriès, 2010	ca. 310	Guangxi, Huanjiang County, Cave Xialan Dong, caves Shui Dong and Shenglong Dong (Golovatch et al. 2010a)
313. *P. bedosae* Golovatch, Geoffroy & Mauriès, 2010	ca. 310	Guangxi, Huanjiang County, caves Dongtu Dong, Huoka Dong and Ganxiao Dong (Golovatch et al. 2010a)
314. *P. bifidus* Golovatch & Geoffroy, 2014	ca. 495	Guangxi, near Fengshan County, Cave Henglixin Dong (Golovatch and Geoffroy 2014, Liu and Golovatch 2019)
315. *P. martensi* Golovatch & Geoffroy, 2006	ca. 1495	Guizhou, Dafang County, Cave Hei Dong; Qianxi County, Honglin Town, caves Luoshui Dong and Luosai Dong (Golovatch and Geoffroy 2006, Golovatch et al. 2007, Liu and Golovatch 2019)
316. *P. sinesis* (Golovatch & Hoffman, 1989)	ca. 1285	Guizhou, Zhenning County, Cave Kaikou Dong (Loksa 1960, Golovatch and Hoffman 1989, Chen and Meng 1990, Liu and Golovatch 2019)
317. *P. superdraco* Golovatch, Geoffroy & Mauriès, 2007	ca. 410	Guizhou, Libo County, Cave Laitai Dong (Golovatch et al. 2007)
318. *P. tiani* Golovatch, Geoffroy & Mauriès, 2010	ca. 310	Guangxi, Huanjiang County, caves Ganglai Dong I and II (Golovatch et al. 2010a)
319. *P. trifidus* Golovatch & Geoffroy, 2014	ca. 180	Guangxi, Guilin City, Cave Kulou Dong (Golovatch and Geoffroy 2014); Yangshuo County, Cave Guanshan No. 4; Xiufeng District, Cave Maomaotou; Yangshuo County, Cave Shangshuiyan (Liu and Wynne 2019)
320. *P. trilobatus* Liu & Golovatch, 2020	ca. 1315	Yunnan, Wenshan County, Liujing Town, Laozhai village, Cave I Dong (Liu and Golovatch 2020)
321. *P. uncatus* Liu & Golovatch, 2020	ca. 1865	Yunnan, Qujing City, Zhanyi County, Cave Tianshengqiao Dong (Liu and Golovatch 2020)
322. *P. whitteni* Liu & Golovatch, 2020	ca. 755	Guangxi, Fengshan County, Jinya Town, Hangdong village, Cave I Dong (Liu and Golovatch 2020)
Genus *Polydesmus* Latreille, 1802–03		Amphi-Palaearctic
323. *Polydesmus liber* Golovatch, 1991	ca. 140	Hong Kong (Golovatch 1991a)
**Family Pyrgodesmidae Silvestri, 1896**		Pantropical
Genus *Cryptocorypha* Attems, 1907		Old World, up to Melanesia in the east
324. *Cryptocorypha spinicoronata* (Zhang & Li, 1981)	ca. 1110	Guangxi, Tianlin County, Langping Town (Zhang and Li 1981b)
**Family Xystodesmidae Cook, 1895**		Holarctic, E and SE Asia up to N Vietnam in the south
Genus *Kiulinga* Hoffman, 1956	10–1080	China
325. *Kiulinga jeekeli* Hoffman, 1956	1080	Jiangxi, Jiujiang City, Jiguling (Hoffman 1956, Zhang and Mao 1984)
326. *K. lacustris* (Pocock, 1895)	10	Zhejiang, 25 mi S of Ninghsien, Lake Wo-Lee (Hoffman 1956)
327. *K. lobosa* Zhang & Mao, 1984	ca. 30	Zhejiang, Zhoushan City, Daishan Island (Zhang and Mao 1984)
Genus *Riukiaria* Attems, 1938	170–4440	E Asia up to N Vietnam in the south
328. *Riukiaria belousovi* Golovatch, 2014	4100	Sichuan, Muli County, SW of Wulaxixiang (Golovatch 2014d)
329. *R. capaca* Wang & Zhang, 1993	170	Fujian, Jiangle County (Wang and Zhang 1993b)
330. *R. chinensis* Tanabe, Ishii & Yin, 1996	885	Zhejiang, Mt. Tianmu (Tanabe et al. 1996)
331. *R. davidiani* Golovatch, 2014	2810	Sichuan, Lixian County, SW of Tonghua (Golovatch 2014d)
332. *R. kabaki* Golovatch, 2014	4440	Sichuan, Kangding City, NNE of Walaxiang, NE of Yusicun (Golovatch 2014d)
333. *R. korolevi* Golovatch, 2014	2900	Sichuan, W of Jiuzhaigou (Golovatch 2014d)
334. *R. martensi* Golovatch, 2014	1700	Shaanxi, Mt. Taibaishan, southern slopes, above Houzhenzi, primary broadleaved forest (Golovatch 2014d)
335. *R. spatuliformis* Golovatch, 2015	2525	Sichuan, N of Luding City, N of Lanan (Golovatch 2015b)
336. *R. tianmu* (Tanabe, Ishii & Yin, 1996)	885	Zhejiang, Mt. Tianmu (Tanabe et al. 1996, Golovatch 2014d)
**Family Opisotretidae Hoffman, 1980**		Himalaya, Myanmar, Indochina, Indonesia, New Guinea, Ryukyu Islands, Japan and Christmas Island, Australia, Indian Ocean (Golovatch et al. 2013)
Genus *Carlotretus* Hoffman, 1980		S China and Sumatra, Indonesia (Golovatch et al. 2013)
337. *Carlotretus triramus* Golovatch, Geoffroy, Stoev & VandenSpiegel, 2013	ca. 200	Guangxi, Chongzuo City, Longzhou County, Shanglong Town, Nonggang Forest (Golovatch et al. 2013)
Genus *Martensodesmus* Golovatch, 1987	150–200	Himalaya, Indochina and S China (Golovatch et al. 2013)
338. *Martensodesmus bedosae* Golovatch, Geoffroy, Stoev & VandenSpiegel, 2013	ca. 150	Guangxi, Hechi City, Du’an County, Baling karst hill (Golovatch et al. 2013)
339. *M. spiniger* Golovatch, Geoffroy, Stoev & VandenSpiegel, 2013	ca. 200	Guangxi, Chongzuo City, Longzhou County, Shanglong Town, Nonggang Forest (Golovatch et al. 2013)

As noted above, according to the ordinal and supra-ordinal distributions in the Diplopoda and a purely biogeographic reconstruction of their origins and early evolution by Shelley and Golovatch (2011), the Oriental Region is the only biogeographic realm of the globe that supports all 16 extant orders of the class. Amongst them, eleven orders are known to occur in mainland China, with the distribution patterns of their constituent families and genera available in Table 1. The remaining five orders, albeit formally excluded from consideration, are added to the roster (Table 2), because representatives of the orders Glomeridesmida, Siphonophorida, Siphonocryptida, Siphoniulida, and Stemmiulida occur or occurred in the adjacent parts of East, Southeast and/or Central Asia. Thus, one extant species of Glomeridesmida and Siphonocryptida each is known from northern Thailand and Taiwan, respectively (Shelley and Golovatch 2011, Golovatch 2015a), several Siphonophorida have been recorded from Vietnam, Laos and northern Pakistan (Jeekel 2001), while fossil Siphoniulida have recently been described from northern Myanmar (Liu et al. 2017c). Two very small orders, Siphoniulida and Siphonocryptida, are considered relict, in a stage of evolutionary decline, whereas most if not all of the remaining orders of Diplopoda are far more diverse and currently in an expansive stage of their evolution (Shelley and Golovatch 2011, Shelley 2011, Golovatch 2015a).

**Table 2.** Distribution patterns of all 16 extant millipede orders, those presently known to occur in mainland China being marked with an asterisk.

Orders	Distribution pattern	Orders	Distribution pattern
Polyxenida*	Cosmopolitan	Siphonophorida	Pantropical
Glomeridesmida	Pantropical	Chordeumatida*	Holarctic + Neotropical + Oriental
Glomerida*	Holarctic + Oriental	Callipodida*	Holarctic + Oriental
Sphaerotheriida*	Old World	Julida*	Holarctic + Oriental
Platydesmida*	Subcosmopolitan	Stemmiulida	Pantropical
Polyzoniida*	Subcosmopolitan	Spirostreptida*	Pantropical
Siphoniulida	Neotropical + Oriental	Spirobolida*	Pantropical
Siphonocryptida	Palaearctic + Oriental	Polydesmida*	Cosmopolitan

The greatest and about equal shares in the diplopod fauna of mainland China expectedly belong to Holarctic/Palaearctic or Oriental elements, with the former naturally dominating the northern, the latter the southern, parts of the country, and both thoroughly mixed and intermingled mainly in the more central parts. The orders Polyxenida, Polyzoniida, Platydesmida, Glomerida, Callipodida, Chordeumatida, and Julida, the families Polydesmidae and Xystodesmidae, as well as certain genera of Paradoxosomatidae seem best to be attributed to Holarctic/Palaearctic components in the fauna of China. In contrast, most of the remaining higher taxa such as the largely tropical orders Sphaerotheriida, Spirobolida, and Spirostreptida, the families Cryptodesmidae, Haplodesmidae, Opisotretidae, and Pyrgodesmidae, as well as several genera of Paradoxosomatidae seem to represent the Oriental stem. Only two families (of 25, or 8%) are endemic or subendemic to China: the monobasic Guizhousomatidae (Chordeumatida), an apparently relict troglobiont from Guizhou Province, and the Paracortinidae (Callipodida) with two genera (maybe just one, see Stoev and Geoffroy 2004) and a handful of species (including two from northern Vietnam). The number of endemic genera is quite high, 16 (of 65, or ca. 25 %): *Sinostemmiulus* (Julida), *Parabilingulus*, *Agaricogonopus*, *Junceustreptus*, *Prominulostreptus* (all Spirostreptida), *Lipseuma* (Chordeumatida), *Angulifemur* (Callipodida), *Belousoviella*, *Gonobelus*, *Mandarinopus*, *Orthomorphella*, *Sigipinius*, *Sinomorpha*, *Wulingina*, *Yuennanina* (all Polydesmida: Paradoxosomatidae), and *Kiulinga* (Polydesmida: Xystodesmidae). One might think the higher the altitude, the more likely the taxon’s Holarctic or Palaearctic origin and, *vice versa*, the lower the elevation, the more probable a tropical descent. However, the vertical distributions usually fail to provide a clear-cut support to attributing a higher taxon to this or that stem. The following examples can serve to show this.

The huge, Eurasian, warm-temperate to tropical genus *Hyleoglomeris* (Glomeridae, Glomerida) currently contains 100+ species, including numerous cavernicoles. Unlike the glomerid fauna of the adjacent Indochina which harbours a considerable proportion of endemic genera (60 % in Vietnam), continental China currently supports only 32 species of *Hyleoglomeris*, most of which occur in caves alone (Golovatch 2015a). The genus ranges from the Balkans in the west, though Anatolia, the Caucasus, Central Asia, the Himalaya, Myanmar and Indochina, to Taiwan, the Philippines and Sulawesi, Indonesia in the east. Importantly, a fossil congener is known from Baltic amber (Eocene, 44 Mya) (Wesener et al. 2019). *Hyleoglomeris* spp. are widespread across China and occur at various elevations, from nearly sea-level to high mountains (Fig. 2), the highest record belonging to *H. sinensis* (2810 m a.s.l.) (Table 1). In the Himalaya of Nepal, one species occurs even higher in the mountains, being high-montane: *H. khumbua* Golovatch, 1987 (3250–3300 m a.s.l.) (Golovatch and Martens 2018).

A very similar pattern is demonstrated by the subendemic genus *Paracortina* (Paracortinidae, Callipodida), with 12 species, of which ten (Fig. 3) are confined to the mountains of southwestern China (Liu and Tian 2015c), mostly high-montane (3300 m a.s.l., Table 1). Only a few are cavernicoles.

*Nepalmatoiulus* (Julidae, Julida) is another very large genus which presently comprises 55 species that span from the central Himalaya in the west, through Bhutan, Myanmar, Indochina, Thailand and West Malaysia, to the Ryukyus, Japan and Taiwan in the east (Enghoff 1987b). Seven species range across the southern parts of China (Fig. 4), including two high-montane ones (2750–3650 m a.s.l., Table 1). Although Beron (2008) reported closer unidentified Diplopoda from up to 5300 m a.s.l. from Nepal, the world’s highest record for a known species belongs to *N. ivanloebli* Enghoff, 1987, also from Nepal: 4800 m a.s.l. (Enghoff 1987b, Shelley and Golovatch 2011). The same general pattern is observed in the similarly speciose (ca. 50 spp.), but more boreal genus *Anaulaciulus* (Julidae), the distribution of which covers northern Pakistan and India, the Himalaya, northern Myanmar, the Far East of Russia, all Japan and Korea, Taiwan, as well as central and eastern China. The highest record belongs to *A. bilineatus* Korsós, 2001 from Nepal: 3600–4300 m a.s.l. (Korsós 2001). Unlike *Nepalmatoiulus*, no *Anaulaciulus* spp. are known to occur in southern China, both these genera being allo- to parapatric. Among the Julidae in China, only very few are cavernicoles.

Particularly clear Palaearctic origins are observed in the large genus *Skleroprotopus* (Mongoliulidae, Julida), most species of which inhabit the Russian Far East, Korea, Japan and China (Table 1), the small Siberian genus *Angarozonium* (Polyzoniidae, Polyzoniida) only marginally encountered in northern China (Table 1), the rather small Siberio-Nearctic genus *Orinisobates* (Nemasomatidae, Julida) represented in China by a single species endemic to the southern Tianshan Mountains (Table 1) (Mikhaljova 2017). The same concerns *Polydesmus* (Polydesmidae, Polydesmida), a very large genus with ca. 80 species, most of which occur in Europe, the Mediterranean area, Anatolia and the western Caucasus, but a few are known from Japan, and one each in northern Vietnam and Hong Kong (Table 1) (Golovatch 1991a, Nguyen 2009).

The large genus *Nepalella* (Megalotylidae, Chordeumatida), with its 27 species spanning from Nepal (10 species) in the west, through Myanmar (two species) and Thailand (two species), to Vietnam (one species) in the south, and southwestern China (12 species, including several presumed troglobionts) in the north (Liu, Wesener et al. 2017d), shows the same general pattern (Fig. 5). Most congeners are mid-montane, but one, *N. marmorata*, has been recorded from ca. 4350 m a.s.l. (Table 1).

Basically the same picture is revealed in the distribution of the huge Central to East Asian genus *Epanerchodus* (Polydesmidae, Polydesmida) which presently encompasses 118 species or subspecies, both epi- and endogean, including 25 across almost entire continental China (Liu and Golovatch 2018b) (Table 1, Fig. 6). Their vertical distributions range from nearly sea-level to high-montane (3090 m a.s.l.), but a few congeners from the Himalaya occur even up to 4250 m a.s.l. (Golovatch and Martens 2018).

The genus *Pacidesmus* (Polydesmidae, Polydesmida) shows a highly peculiar distribution (Fig. 7), with all of its eleven Chinese species being low- to mid-montane and restricted to karst caves in the south (Liu and Golovatch in press), whereas the type species, *P. shelleyi* Golovatch, 1991, comes from the summit (2200–2500 m a.s.l.) of Mount Doi Inthanon, northern Thailand (Golovatch 1991a). Similarly, the small genus *Glenniea* (Polydesmidae) contains five lowland to mid-montane epigean species from the Himalaya of India and Bhutan (Golovatch and Martens 2018), as well as another three species (including two cavernicoles) from southern China (Golovatch and Geoffroy 2014) (Table 1, Fig. 8).

The great Holarctic family Xystodesmidae (Polydesmida) presently encompasses 66 genera and ca. 410 species, most of which occur in the Nearctic. Only few genera and species are known from Central and northern South America (to Ecuador in the south), the Antilles, the Mediterranean region and East Asia (Shelley and Smith 2018). The largest East Asian genus *Riukiaria* currently contains 35 species or subspecies from southern Japan, southern Korea, Taiwan and China (Korsós et al. 2011, Golovatch 2014d, 2015b, Nguyen 2016). We disagree with Nguyen (2016), who split *Riukiaria* into two genera and created a new genus, *Parariukiaria* Nguyen, 2016, to accommodate a new species from northern Vietnam and three previously described ones from China. To our mind, *Riukiaria* and *Parariukiaria* show all transitional stages in the reduction of a gonoprefemoral process and, albeit without formal synonymy advanced here, both may well be regarded as representing a single large genus, in which several peripheral, southernmost congeners demonstrate a more or less strongly suppressed process on the gonopodal prefemur, from relatively small to totally missing. All nine *Riukiaria* species in China are epigean and span across the central and southern parts of the country, occurring in lowland to high-montane habitats (170–4440 m a.s.l., Table 1, Fig. 9).

As noted above, in China the great family Paradoxosomatidae, which is amongst the largest in the class (200+ genera, 1,000+ species), dominates most of the tropical faunas across the world, but is absent from the Nearctic, contains remarkably few troglobionts (Golovatch 2015a) and comprises genera of various origins. Some seem to be rooted in the Palaearctic (including several endemic or subendemic ones), the others are likely to be Oriental. Among the former elements, the following two rather species-rich genera can be taken as examples.

The genus *Hedinomorpha* is subendemic to China, with most of its 17 species known from the country being high-montane (up to 4490 m a.s.l., Table 1, Fig. 10), and only one more restricted to Tajikistan, Central Asia (Golovatch 2019b). The genus *Sigipinius* is strictly endemic to mainland China and contains nine high-montane species (2810–4195 m a.s.l., Table 1, Fig. 11). Such paradoxosomatid genera as *Cawjeekelia*, *Kronopolites*, *Mandarinopus* and *Orthomorphella* likewise seem best to be attributed to Palaearctic elements in the fauna of China.

In contrast, Paradoxosomatidae also contain a good number of presumed Oriental components, mostly tropical to subtropical. Thus, the genus *Hylomus* presently comprises 36 species from Myanmar, Thailand, Laos, Vietnam and China (Srisonchai et al. 2018, Liu and Wynne 2019, Golovatch 2019b). Many of them are presumed troglobionts. The distributions of all 20 *Hylomus* spp. recorded from China cover much of the southern and eastern parts of the country and are only confined to lowland to mid-montane habitats (ca. 140–910 m a.s.l., Table 1, Fig. 12). At the moment, with its 73 species (Golovatch 2019b) that range from southern China in the north, through most of Indochina, to Myanmar in the south, *Tylopus* remains the largest genus of Paradoxosomatidae globally. However, the altitudinal distributions vary from lowland to high-montane (350–4025 m a.s.l., Table 1), cavernicoles are few, while the Chinese congeners mark the northern range limit of the genus and are confined to the southwestern parts of the country (Fig. 13). Because *Tylopus* and *Hedinomorpha* seem to be particularly similar morphologically and co-occur, albeit probably never strictly sympatric, in southwestern China (at least Yunnan, Figs 10, 13), these areas seem to mark the southern range limit of *Hedinomorpha*.

The relatively large genera *Anoplodesmus*, *Antheromorpha*, *Enghoffosoma*, *Nedyopus* and *Sellanucheza* also seem best to refer to as Oriental components in the fauna of China, because it is southern China that marks their northern range limits. The same concerns the small genera *Hirtodrepanum*, *Inversispina*, *Piccola*, *Polylobosoma* and *Tetracentrosternus*, all of which show one or a few congeners either in the Himalaya and/or Myanmar, or northern Vietnam, or Taiwan. The mono- or oligotypic *Belousoviella*, *Gonobelus*, *Sinomorpha*, *Wulingina*, and *Yuennanina* are all strictly endemic to China, mostly to its southwestern parts, but their Oriental stem is clear-cut due to their closest affinities.

The immediately above paradoxosomatid genera endemic or subendemic to southern China which all seem to be of Oriental stock, together with some other polydesmidans like *Carlotretus* and *Martensodesmus* (both Opisotretidae), *Glenniea* and *Pacidesmus* (both Polydesmidae, Figs 7, 8), as well as several others (e.g. Cryptodesmidae, Haplodesmidae, Pyrgodesmidae), regardless of whether they are Oriental or Palaearctic in origin, seem to be sufficiently numerous and manifest to warrant the recognition of a separate, albeit secondary, subordinate, southern Chinese diversity and faunogenetic centre which must have seriously contributed to at least the faunas of the adjacent parts of the Himalaya, Myanmar, Thailand, Indochina and Taiwan. The influence of that southern Chinese centre in the Himalaya has recently been emphasized (Golovatch and Martens 2018).

The Oriental realm as one of the main sources for the formation of the millipede fauna of China can also be exemplified by the basically tropical to subtropical orders Sphaerotheriida, Spirobolida and Spirostreptida, as well as the families Cryptodesmidae, Haplodesmidae, Opisotretidae, Pyrgodesmidae (all Polydesmida) and Sinocallipodidae (Callipodida), some of which often vary a lot in altitudinal distributions just like numerous Holarctic/Palaearctic groups. The often presumed rule “tropical elements for low elevations only” does not always work.

The genus *Glyphiulus*, the largest in the family Cambalopsidae (Spirostreptida), presently comprises 60+ species in East and Southeast Asia (to Borneo in the east), 42 of which are encountered at 105–4150 m a.s.l. across China (Fig. 14). Most of them are cavernicoles (Liu and Wynne 2019). A similarly large and even more widespread genus, *Eutrichodesmus* (Haplodesmidae), presently encompasses 50 species (Liu et al. 2017b, Liu and Wynne 2019) which range from southern Japan and Taiwan in the north, through entire Southeast Asia, to Vanuatu, Melanesia in the south. The distributions of all 24 species that populate continental China seem to be more typical, much better agreeing with the above rule: 65–1495 m a.s.l. (Table 1, Fig. 15). At least half of them are also cavernicoles.

## Discussion

The diversity estimates presented in Table 1, i.e. 339 species, 71 genera, 26 families, and eleven orders, are much or significantly higher than those reported from the main adjacent areas. The similarly huge territories of Siberia and the Russian Far East that lie north of China support only ca. 130 species, 46 genera, 18 families and five orders of Diplopoda, while the fauna is reasonably well known (Mikhaljova 2017). This is hardly surprising because the prevailing permafrost and sharply continental climates of Asian Russia are largely too harsh to sustain a rich millipede fauna. The even harsher, mostly arid Mongolia is extremely poor in millipedes, with some nine species, five genera and families, and three orders involved (Mikhaljova 2012, Nefediev et al. 2015).

In contrast, the great Himalayan Range spanning for >2,300 km from northwest to southeast and mostly lying south of China supports >275 species, 53 genera, 23 families and 13 orders of diplopods (Golovatch and Martens 2018). Similarly, the fauna of India presently amounts to > 270 species, at least 90 genera, 25 families, and eleven orders (Golovatch and Wesener 2016), *vs.* 92 species from 34 genera, 13 families, and eight orders recorded from Myanmar (Likhitrakarn et al. 2017) or ca. 230 species in Thailand (Likhitrakarn et al. 2019). A direct correlation between area and latitude is clear: the larger the area and the closer it lies to the equator, the richer the biota, including the diplopod faunas. However, the more southerly, the greater the diversity, and the more incomplete and fragmentary is our knowledge.

Certainly the Chinese millipede fauna still remains strongly understudied, given the country’s great size and habitat diversity, including the globe’s greatest karst areas. It may well amount to 1,000 species (Golovatch 2015a), chiefly due to the still particularly poorly studied micropolydesmidans, as well as cavernicoles. Southern China’s karsts are unique in often harbouring up to 5–6 diplopod species per cave (Golovatch 2015a). At least some of the remaining orders such as Glomeridesmida, Siphonocryptida, Siphonophorida, Siphoniulida, and Stemmiulida that occur in the Oriental Region (Table 2), including areas immediately adjacent to mainland China, may also be expected to populate the country. For example, Jiang et al. (2019) have recently described a fossil Siphonophorida from Cretaceous amber (ca. 99 Mya) in northern Myanmar, and an extant species is long known to occur in northern Pakistan (Golovatch 1991b). In addition, the same Burmese amber contains still undescribed Stemmiulida (Stoev et al. 2019) and two described species of Siphoniulida (Liu et al. 2017c). Likewise, as noted above, an extant species of Siphonocryptida and Glomeridesmida each is known from Taiwan and northern Thailand, respectively (Korsós et al. 2008, Shelley 2011).

While the Palaearctic/Holarctic components expectedly dominate the fauna of the northern parts of the country, the Oriental ones prevail in its south and along the Pacific coast. Both realms are increasingly mixed and intermingled towards China’s centre. However, in addition to the above traditional views, based on millipede distribution patterns alone, southern China seems to harbour a subordinate, but highly peculiar faunal nucleus, or origin centre of its own, whence the adjacent Himalaya, Indochina and/or Taiwan could have become populated by younger lineages. The presence of a family (the monobasic Guizhousomatidae) and numerous genera endemic or subendemic to southern China, both apparently relict and relatively advanced, seems to be evidence of this. Within the order Callipodida alone, the family Sinocallipodidae seems to be the basalmost and representing a suborder of its own, the Paracortinidae is a more advanced subendemic, same as the mostly Central Asian Caspiopetalidae (Stoev and Geoffroy 2004, Stoev and Enghoff 2011). More importantly, a fossil family representing a separate suborder has recently been discovered in the Cretaceous Burmese amber, ca. 99 Mya (Stoev et al. 2019).

The millipede fauna of mainland China is thus a tangled mixture of zoogeographic elements of various origins and ages, apparently both relict and more advanced. The few anthropochores/introductions must have been the latest faunal “layer” to populate China.
